# What Next for Trauma-Informed Education Research? A Research Prioritisation Exercise with Young People as Informants

**DOI:** 10.1007/s40653-025-00711-3

**Published:** 2025-05-23

**Authors:** Eleanor F. Bryant, Darren Moore, Abigail Emma Russell

**Affiliations:** 1 Children and Young People’s Mental Health Research Collaboration, Department of Public Health and Sports Sciences, Medical School, University of Exeter, University of Exeter, Exeter, UK; 2 Graduate School of Education, University of Exeter, Exeter, UK

**Keywords:** Research prioritisation, Childhood adversity, Adverse childhood experiences, Lived experience, Trauma-informed practice, Children and young people

## Abstract

Children and young people (henceforth referred to as children) who experience severe childhood adversity are at risk of mental health and developmental challenges that mean standard approaches to education are not able to support their needs, resulting in high levels of exclusion and alternate provision. Evidence of the best education models to support positive outcomes is needed. This exercise aimed to identify research priorities of children impacted by severe adversity and the people who surround/support them to be used to inform future work surrounding the education of children with a history of trauma. Research prioritisation methods were used. The views of adult stakeholders with lived or professional experience, and children with histories of trauma who attend a specialist school were collected. Two surveys were circulated in each group collecting: 1. Respondents’ questions (priorities) about education for children with trauma; 2. Rankings of these priorities. The first children's survey was carried out within a school. The resulting 25 questions were presented to eight students who each chose three questions which they deemed most important. In the adult survey, 114 respondents suggested 196 questions. The children's questions were combined with the adult questions which resulted in 221 questions in total. These were categorised into six themes (Child Centred, Home, Interventions and Alternate Provisions, Policy & Wider, School, and Teachers & Learning). Questions were re-circulated under these categories, and 48 individuals ranked their top 5 or 10 questions (dependent on the number of questions in the category), and the top five questions overall were labelled as priorities. The top priorities generally reflected well-researched areas in the field of trauma, namely how trauma-informed learning and care can be implemented in mainstream schools. This suggests a lack of evidence communication. Future research should aim to improve knowledge dissemination.

**Supplementary Information:**

The online version contains supplementary material available at 10.1007/s40653-025-00711-3.

## Introduction

A groundbreaking study carried out in 1998 found that as many as half of adults report having experienced an adverse childhood experience (ACE) (Felitti et al., 1998). ACEs are potentially traumatising events that occur during childhood and adolescence and include exposure to violence in childhood (physical, sexual or psychological) or witnessing potentially traumatic experiences in the home (e.g. intimate partner violence, mental illness, or substance misuse) (Merrick et al., 2019). It is difficult to define at what stage ACEs become trauma, which can be defined as “an emotional shock that may have long-term effects on behaviour or personality” (Chambers Concise Dictionary, 2004), and indeed this will vary between individuals as well as depending on the severity, proximity and chronicity of adversity experienced. Research has shown that a significant minority (around 15%) of children develop PTSD after trauma exposure (Alisic et al., 2014), and exposure to any ACE is associated with almost three times the odds of depressive symptoms (Cheong et al., 2017). This indicates that experiencing adversity in childhood can impact individuals throughout adolescence and into adulthood. Traumatic experiences have also been shown to affect child brain development (Anda et al., 2006; Child Welfare Information Gateway, 2011; Perfect et al., 2016), is associated with problems in mental and physical health (Felitti et al., 1998), and school life through academic performance (Hurt et al., 2001; Purtle, 2020; Stein et al., 2003), as well as cognitively, socially and emotionally (Perfect et al., 2016). It is important to understand the mechanisms that lead to these deficits in order to improve outcomes, and to help young people achieve their goals.

Education is a critical area to focus on for children and young people with challenging pasts. Many barriers face a student with trauma when attending school – differences in cognition and short-term memory inevitably lead to problems with educational attainment (Bremner, 2006; Douglas Bremner et al., 1995). Moreover, behavioural problems (Bruce, 2010; Fitzpatrick & Boldizar, 1993; Martinez & Richters, 1993) and hypersensitivities or hyperarousal (Brunzell et al., 2019) may cause difficulties in over- or under-stimulating schooling environments.

It has been suggested that trauma sensitive practices should encompass two separately proposed ideals of teaching. Firstly, *trauma-informed practice* which puts healing at the forefront; Secondly, *wellbeing-informed practice* which prioritises growth (Brunzell et al., 2019). Although the societal and cultural goal of school is educational attainment, in order for a child to reach their educational potential, they need to be mentally and physically well (Murphy et al., 2015). Therefore, education that is trauma sensitive may need to prioritise mental health and functional skills before academic subjects.

As we continue to research how we can improve outcomes (such as wellbeing and educational attainment) through teaching practices, it is crucial that this research addresses the needs of the population. One way to assess gaps in academic literature is through Patient and Public Involvement (PPI) in research (Grotz et al., 2020). PPI can include people in the research cycle at many different stages, with ‘identifying and prioritising research’ often seen as a first step to creating a wholly inclusive research programme. This is vitally important if research is to benefit those which it aims to, and can help those taking part in the PPI during the process (Brett et al., 2014). Research prioritisation exercises produce a clear and useful outcome. 

Research prioritisation utilising the involvement of young people is becoming increasingly common, especially in the field of youth mental health, and yet no research prioritisation has been undertaken in the area of trauma and education. Additionally, much of the existing research is school-based. Therefore, we deemed it important to consult ‘experts by lived experience’ who might have expertise outside of the mainstream school setting (Avery et al., 2021).

This exercise set out to try and establish the priorities and everyday realities of education of trauma-experienced children. We aimed to find out:

1. What the research priorities were for children and adults that gained consensus
2. Whether areas more-comprehensively researched in the literature are truly the priorities of the community, or if other new priorities and opportunities for future research are emerging
3. Whether the priorities set by young people match those set by important adults in their lives
4. The level of knowledge of the scientific literature within the wider community

Many established methods exist for the process of research prioritisation, for example, that set out by the James Lind Alliance. However, these have a highly structured format which do not allow for the freedom sometimes required to achieve a project’s goals (Forbes et al., 2022). In this project, a simple methodology was created drawing features from many other research prioritisation studies (Forbes et al., 2022; Tikellis et al., 2021), as described below. There were two target groups for the prioritisation, one being experts by experience who self-defined as having ‘lived’ or ‘professional’ experiences (parents, carers or relatives, school-based professionals, members of a community or charity, and other related professionals). The other group were children enrolled in a school that specialises in taking students who have an Education and Health Care Plan with Social, Emotional, and Mental Health as the primary need, and have difficult backstories. The language we have used throughout this article reflects that used by these two groups, and discussions with the school staff about the terms preferred by their students.

## Participants and Methods

### Participant Information

Adult stakeholders with links to this topic may have a limited range of specific qualifications or job roles. Subsequently, we did not define eligibility criteria for this study based on training background or job role. We relied on their knowledge from a range of experiences, including in schools, with family, or as a member of a training organisation. Participants completed a mandatory question ‘what is your role in relation to this topic?’ and our recruitment strategy (below) targeted those likely to have relevant professional experience with children with complex backstories. All survey responses were deemed as a suitable basis to have either lived or professional knowledge. Eligibility to take part in the second stage of either survey was independent of having taken part in the first stage.

Reimbursement was not provided for any respondents to the adult stakeholder survey, and was not provided directly to the children that took part in the survey, but was provided to the school the students attend, in the form of outdoor play equipment to the value of £80. This was not known by school staff previous to the implementation of the survey. Research prioritisation does not require research ethics approval, however we were mindful of conducting this exercise in a safe and ethical manner, so in order to elicit young people’s voices we collaborated closely with one specialist school. We deliberately do not report many details about the school in order to maintain confidentiality.

### Methods Overview

Four surveys were carried out in total in this Delphi study design. Children with a history of trauma were contacted via a specialist school, where staff members asked the students what questions they had about their education. These questions were then presented to the students in a list to choose which they were most interested in using a tally. Staff at the school who were involved in either of the surveys were invited to a focus group after data collection had finished to collect their views on the results that were collected from both surveys. Key adults were targeted with a survey to suggest research questions that they had surrounding education of children with a history of trauma. These were grouped into six themes, and the second survey was recirculated for the same groups to prioritise the questions using a vote counting method.

#### First Children and Young People’s Survey Participants and Methods

The questionnaire was carried out in one specialist school in the South West of England. It was initially trialled with one student from year 10 and then carried out with five classes at the school ranging from year 6 to year 10 and ages 5–16 years old (although the age of students in the classes does not necessarily correspond to year groups). Teachers and school staff assisted children on the task after the authors discussed with the school mental health practitioner about the most appropriate way to conduct the research prioritisation with students. Staff the students knew and trusted were deemed most able to support them with difficult topics which may occur during the discussion about their top research questions. The school mental health practitioner was provided with an information sheet to assist the staff to prompt questions from the children and young people. Supplementary Information 1 shows the recruitment poster, overview of the steps of research prioritisation, and the staff briefing sheet which were all supplied to the school mental health practitioner. Questions were communicated by the school mental health practitioner by email to EB.

#### Analysis of First Children and Young People’s Survey

The relatively small sample and number of questions required little analysis. All of the questions submitted were included in the next round of the children survey, but in some cases were rephrased from first person to the third person perspective.

#### Second Children and Young People’s Survey Participants and Methods

Children that took part in the second part of the process were attendees at the same school as above, including some different students. The students were presented with a list of all the questions that had been submitted in round 1 by students, and asked to choose the top three that they thought were “most important to do research on”. This stage of the adapted Delphi process, like in the first stage of the survey, was administered in class by teachers and classroom staff. Although the young people’s questions were included on the adult’s survey, we chose not to include the adult’s questions in the second stage of the child’s Delphi. This decision was made based on discussion with school staff and the content and number of the questions.

#### Analysis of Second Children and Young People Survey

The top two ‘most voted for’ questions were extracted to be used as ‘top priorities’.

#### First Adult Stakeholder Survey Participants and Methods

The survey was circulated through emailing recruitment materials to networks including Adoption UK (*Adoption UK Charity*, n.d.), Plymouth Trauma-Informed Network (*Plymouth Trauma Informed Network – Plymouth Octopus*, n.d.), Touchbase (Formula, n.d.) the Children and Young People's Mental Health Research Collaboration X, formally Twitter account (https://x.com/) and the University of Exeter Graduate School of Education (https://education.exeter.ac.uk/) via email. The organisations were asked to circulate these in the way that worked best for them and their client base. The survey was titled “Submit a Question!”, was made using Microsoft Forms (*Microsoft Forms*, n.d.) and can be seen in its entirety in Supplementary Information 2. The main question asked was “What questions do you think we should be investigating about the education of children with complex or difficult life stories?”. Respondents were provided with some examples of questions to demonstrate potential topics or formats that questions could take (see Supplementary Information 2). Participants were also asked, “Why do you think it is important to get an answer to this question?”. Participants were encouraged to submit multiple questions if they wished to, and the survey could be completed multiple times by the same participant if further questions arose. The questionnaire was open between 31/01/2022—31/03/2022. Participants were given the opportunity to leave an email address if they were interested in being updated about the progress of their question(s) in the exercise; aside from this, the survey was completely anonymous although some participants did name places where they worked.

#### Analysis of the First Adult Stakeholder Survey

Questions were reviewed by EB and any without sufficient detail were incorporated with participants’ answers from the “Why do you think it is important to get an answer to this question?” section of the questionnaire. At this point, the student questions were combined with the adult questions. The language was not changed between the questions that were used on the second children’s survey and the second adult stakeholder survey to preserve the meaning that the original questions posed. We sorted questions into categories due to the high volume of questions received, in order to keep the questionnaire short and reduce burden on respondents.

EB and AR discussed each question, noting aspects of Bronfenbrenner's Ecological systems theory (Bronfenbrenner, 1974). The final category for the question was chosen based on the sample and methods that would be utilised to carry out research on the question. This resulted in six themes which were stored using NVivo (*NVivo*, n.d.) and are described below:

A. **The child.** These were questions which focussed on the experiences of a trauma-experienced young person, possibly with other specific elements, which would be researched by investigating the stories and thoughts of children who have experienced trauma individually to capture the range of experiences, with children who have experienced trauma best-placed to answer these questions on the whole.
B. **Home Life.** These were questions that could be effectively researched with a range of designs or processes, but that all centre on how life outside of school can impact a child’s education, or equally how experiences within school can affect the home life of a student. This category also includes questions about carers who only see the child outside of an educational setting but may still affect the child’s experiences.
C. **Interventions or Alternate Provisions.** This category mainly includes questions which are interested in measuring outcomes (of which a range were highlighted in questions, for example, mental health, academic outcomes, and behavioural changes). Also highlighted were questions about what other approaches exist to assist in classrooms and overcome barriers. Some questions touched upon whether some specific interventions or behavioural techniques were not effective in alternate provision or with this population of students with additional needs, where they may be in mainstream settings.
D. **Policy and Wider Implications.** These questions were placed in this category either due to an explicit link to policy or if they would be best answered by measuring this across a large sample of students within a large number of schools, hence having generalisable conclusions or impacts.
E. **School and the school environment.** Although it may seem clear that all questions about education will be about school, many children who have experienced trauma are placed within alternative education settings that better suit their needs and requirements for effective learning. These questions encompass topics including the physical environment, the wider school staff, and the school's policies. These are questions which would be answered with investigations carried out in individual schools, as they are likely to be heterogeneous in their practices.
F. **Teachers and methods of learning.** This may seem to overlap with the intervention aspect of category three, it is important to distinguish between interventions which often aim to change behaviour and the pedagogical aspect of learning. Several of these questions focus on how the child engages in their learning and what might impede this. Another important factor in learning is the teacher, and many questions are highlighted regarding how their practice can help improve children’s learning experiences, and how their training and personal experiences may affect this.

#### Second Stakeholder Survey Participants and Methods

This survey contained the children’s and adult’s questions, split into the six categories above. It was circulated through the same groups and networks as the first stakeholder survey. Respondents could rank just one or as many of the sections as they liked. Participants could choose their top 10 questions in each of the sections, (with the exception of the ‘home’ category, which had fewer questions overall than other categories, so five were chosen). The survey was available between 11/05/2022 and 08/06/2022. An example of the survey pages can be seen in Supplementary Information 3.

#### Analysis of the Second Stakeholder Survey

From each of the six categories, the top five questions with the most votes were extracted, giving a total of 30 top questions.

#### Focus Group

A focus group with members of the school and classroom staff was undertaken on 14/06/22, one week after the closure of the adult survey. This aim of the group was to hear staff feedback on their perceived accuracy and usefulness of the priority-setting process, how this could be improved in future research, and their opinions on the finalised priorities. A topic guide was followed (see Supplementary Information 4) and the group was recorded, with prior consent from those present, for note-taking purposes only.

## Results

### First Children and Young People’s Survey

The student survey was carried out in five classes at the school, resulting in 25 questions.

### Second Children and Young People’s Survey

Eight young people then took part in the second stage of the process. The list of questions suggested by the students was sent to a staff member at the school and left in an editable form so that the phrasing could be changed if needed due to potentially upsetting terminology. As there were a small number of participants in the second round of the survey, two priorities were identified. The full list of questions can be found in Supplementary Information 5. The top two questions both received three votes and were:

1. Is everyone who’s had difficult life experiences, an overthinker?
2. Why does being on technology help us be calm?

### First Adult Stakeholder Survey

**Fig. 1:**
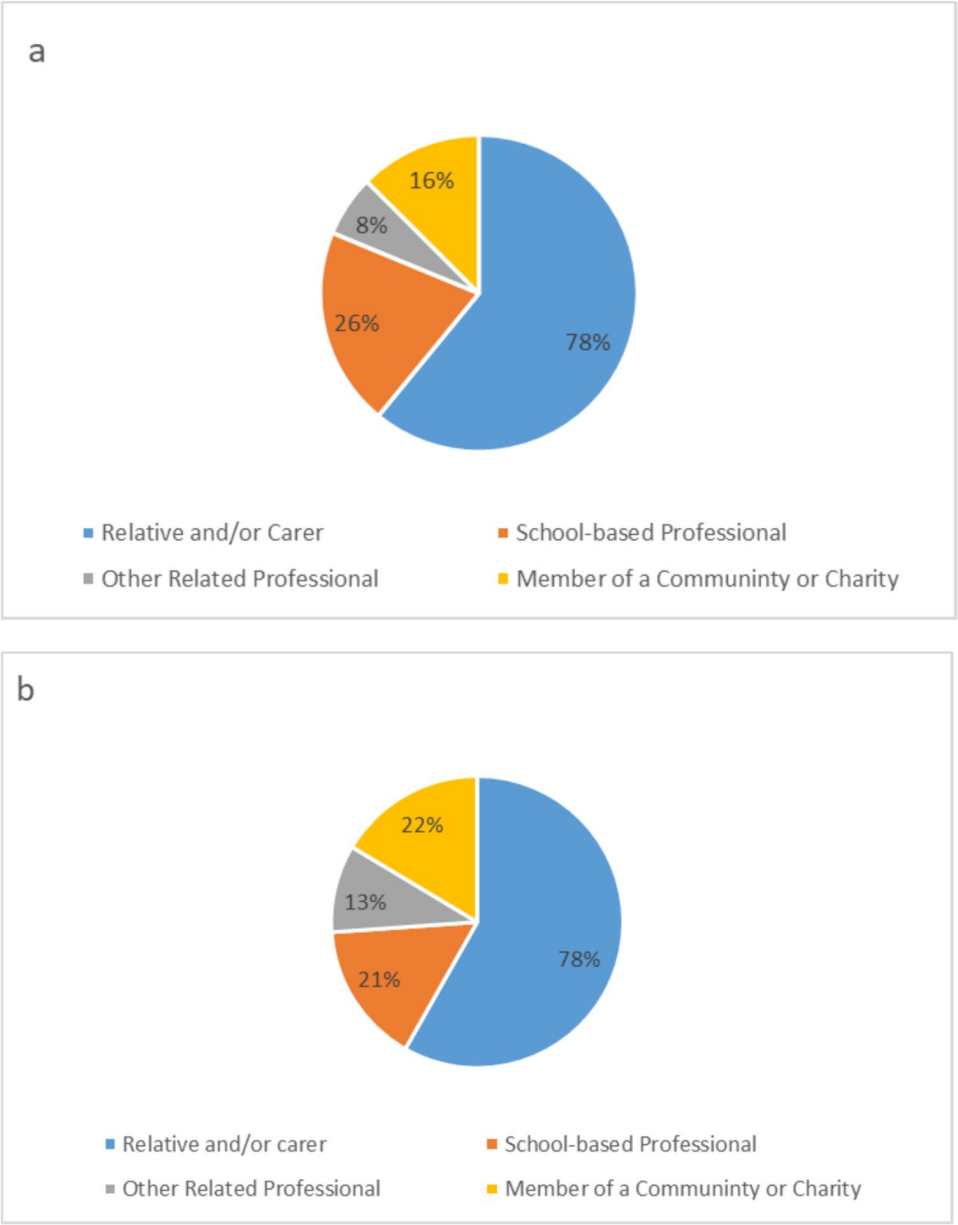
The percentage of each group of respondents that took part in **a** the first adult stakeholder survey circulated **b** the second adult stakeholder survey circulated. The percentages do add up to over 100%, as many participants chose more than one option for their involvement. Because of this some participants were included in multiple categories

There were 114 separate responses to the first survey, with 27 participants submitting more than one question within one submission. It is not known, however, whether any of the participants submitted more than one entry, as all responses were logged anonymously. Figure 1a displays the answers to “What is your role in relation to this topic?”, which fit into four categories. A complete list of roles can be seen in Table 1. This led to a total of 196 questions which were included in the second round of the survey. The 25 responses collected from the children’s questionnaire were combined with these, resulting in 221 questions in total to be included in the second adult questionnaire.

**Table 1:** Breakdown of all respondent types to the first and second adult surveys. Total N. is the number of people in that group that participated across both surveys. % Total is the percentage that that group made up of respondents to both surveys combined

Group	"What is your role in relation to this topic?"	N 1 st survey	N 2nd survey	Total N	% Total
Relative and/or carer	Parent or Carer, Family Member, Homeschooling Parent	89	36	125	78.1
Member of a community or charity		18	10	28	17.5
School-based professional	Teaching staff	19	8	40	25
	Pastoral/SEN Staff	11	2		
Other professional^a^		9	6	15	9.4

^a^Other Professional Includes: Counselling services, Policymaker, Senior Inclusion Manager at Local Authority, Head of education for fostering agency, Support Worker, Play therapist, Trauma recovery practitioner, GP doctor, Mental Health Practitioner, Social Worker, Clinical Psychologist

### Second Adult Stakeholder Survey

The second survey was hosted on online surveys.ac.uk (*Online Surveys*, n.d.). There were a total of 48 responses. In the categories ‘Child Centred, ‘Home’, ‘Interventions and Alternate Provisions’, ‘Policy and Other’, and ‘School’ all 48 participants took part in the survey, ranking at least one of the questions, and 47 participants ranked at least one question in the ‘Teachers and Learning’ category. Figure 1b and Table 1 display the answers to “What is your role in relation to this topic?”.

The top five questions in each category can be found in Supplementary Information 6—questions with an equal number of votes have been given the same number. The most highly ranked question from each category are as follows:

- **Child Centred**: What do trauma responses look like in the classroom?
- **Home:** How can schools work in partnership with families?
- **Interventions and Alternate Provisions:** How can we give teachers tools to respond to traumatised children when they are dysregulated and display inappropriate behaviour in school?
- **Policy and Wider:** What trauma-informed ways of working are there in mainstream education?
- **Schools:** How do schools better meet the needs of children with a trauma history where there is no specific diagnosis?
- **Teachers and Learning:** How can schools improve their teaching methods to be more trauma-informed?

### Focus Group

The focus group was made up of seven members of school staff: three learning support assistants, a mental health practitioner, a pastoral lead, a trauma recovery practitioner and a teacher, and was conducted by EB and AR. In response to the first question “To those that were involved, how did you find the first phase collecting the questions from young people?” it was said that explaining the concept to students was challenging and that teachers themselves did not feel they could explain this despite the information sheet provided. We had initially tried to carry out a whole-staff training meeting, however this was impossible due to time constraints and the workload of the school staff. Because of this, EB met with the mental health practitioner at the school and discussed the process, which she then disseminated to other staff. Students found the activity interesting but it was also inaccessible to some who did not believe themselves to have experienced a traumatic event.

Participants noted that in the second phase students liked seeing their own questions being put into action, and did not have a preference for questions that they had suggested themselves. Nonetheless, the long list of questions was overwhelming to the children, which may have led to a preference for questions at the top of the page.

Staff did note that they were surprised by some of the questions, namely “Why does being on technology help me be calm?” as this contradicted the experiences of some members of the group, but despite this, it is an interesting insight into the childrens’ perspectives. Another example of this surprise is in response to the question “Why do I have a low pain threshold?”. This was something the group had not been aware that students considered previously, and brought some insight. Members present also questioned what the term “overthinker” can mean in response to the question “Is everyone who’s had life experiences make you an overthinker?”. They were unsure what the word “overthinker” was being used to mean, but noted that it was used relatively frequently by students.

Members of the group noted that they had expected more questions to focus on the topic of “consequences” as children in the school often pose questions about the consequences of theirs or others’ actions, and this stood out to the staff as something they had expected to see more of in the prioritised questions.

A main takeaway from the focus group was the need for children to know *why* something works, and for adults and others to know *what* works. The children’s questions were generally considered to be more original and stimulating, as well as more helpful to classroom practice as ultimately the focus group participants felt the children were at the centre of research in this area.

In reflecting on responses to the adult stakeholder surveys, focus group participants noted that a large proportion of survey participants were individuals who would not necessarily see the time a children spent in an educational setting, and this may explain why there are several highly ranked questions that educational professionals with training and experience might know and apply in their practice. Supplementary Information 7 breaks down the top questions for each group (professional, family, other) and shows the differences between the groups’ preferences. As well as this, the participants wondered whether they (as staff members in a specialist school) were more likely to prioritise the children’s questions, whereas school staff from mainstream schools may be more interested in the questions posed by key adults due to their potential lack of access to training and resources.

Suggestions for future PPI carried out in this way are that this activity would have benefited from a member of the research team visiting the school and conducting the activity with the children. Additionally, reducing the number of decisions to be made, for example by condensing the questions into themes, having cards to support ‘this or that’ decisions, or by splitting the decision-making tasks over more sessions would be beneficial to achieve the most accurate responses. Randomly ordering the questions across children would counter ordering effects and was suggested by the focus group.

## Discussion

This priority-setting process lists the top questions from adult stakeholders and children about educating children with a history of trauma. The prioritised questions suggest that adult stakeholders and children have highly disparate views, furthering the case for more children’s views to be taken into account, particularly in research prioritisation methodologies.

The overlap between adult stakeholder questions and the current literature suggests that the research currently being carried out in this area is indeed confronting key issues to those who stand to benefit from applying evidence-based adaptations. For example, the question “ What trauma-informed ways of working are there in mainstream education?” is answered extensively by Souers and Hall, whose book contains 12 chapters on practical strategies to use within the classroom (Souers & Hall, 2016).

However, this lack of translation from research into awareness and potentially practice may also hint at barriers to research implementation at the individual, school and political level.

Some of this knowledge may be being implemented without stakeholders being aware of the evidence base behind actions. School staff (in a specialist school environment) were mindful that many questions the adult stakeholders posed are already occurring in schools, for example teaching methods that can be used in the classroom and alternate methods such as outdoor learning. This is a cause for concern within the field; parental involvement with their education is an important factor to children (Desforges & Abouchaar, 2003) and moreover, key adults need support and training to be best able to support a child with a history of trauma (Trauma Sensitive Practice with Children in Care, 2014). Some of the questions raised build on findings that teachers can also feel some uncertainty about the education of children who have experienced trauma (Alisic, 2012). These issues might be minimised by improved communication with parents and carers of the literature and from schools. Despite this, it is difficult to say how increased communication could be effectively implemented given the growing pressure on teachers (Ferguson, 2019), and so progression in the field of science communication is needed. Wide-reaching dissemination of lay-friendly evidence summaries in both video and written format, circulated through education, parent, and health networks is required to promote evidence-based practices.

### Application in Practice

The parallels between the suggested questions and the current literature may mean that suggested questions could act as a novel basis for the dissemination of research knowledge to stakeholder groups. The majority group in each round of the adult survey were parents and carers, who, according to the focus group, had different knowledge to that of school staff. This could suggest that parent and carer groups would be an auspicious group to approach for knowledge exchange. They appeared to be a highly engaged group, suggesting large numbers of questions, and being well-represented within the sample. Rapid literature reviews using the prioritised Delphi questions as research questions could then be condensed and shared, creating impact with experts by lived experience. This could present an opportunity for two-way knowledge exchange, and discussions could suggest future pathways for trauma-informed education research.

### Strengths

There are a number of strengths to this study. Importantly, obtaining the views of the children. Though there is little national guidance on the use of PPI (Horobin, 2018), this exercise shows the strength of utilising the research prioritisation process as a method of PPI with a novel group that is rarely included as part of the research. The childrens' questions vary from the themes of teachers and schools and delve more into neurological and physical questions about the effects of difficult life experiences on education. The lack of alignment between the adult priorities and the student priorities demonstrates that novel questions can be raised by involving underrepresented groups within research. Moreover, school staff reported positive engagement with the activity by children and no adverse impacts, whether or not they perceived it to be personally relevant.

### Limitations

One of the main limitations of this exercise is the self-selected sample and the narrow breadth of information available on the participants. We lack information about the specific knowledge or experience of the respondents: although this can be assumed through their self-reported roles. These all reported being key individual roles in regards to a child or young person who has experienced trauma, however there is no way to verify the role information or ascertain further details of level of experience and previous engagement with research and evidence. We chose not to ask additional questions in order to minimise the personal data we were collecting as the study was not subject to ethical committee oversight, and to facilitate participation in generating questions in a minimally burdensome manner. It is also unknown how many respondents to the first survey further took part in the second. The second survey was public for less than one month, which may have skewed responses to those who had the time or ability to respond. However, the effect of the same participants taking part in both studies and voting for their own questions would be negated by the high number of votes that the top questions received. A further limitation of the exercise is that in the second adult stakeholder survey the vast majority of respondents voted in response to all areas which may signify a lack of specialist knowledge on specific topics, for example, policies, which could have prevented the questions from representing the current issues within the area. However, this is also a strength in terms of sample size and engagement, as the majority of participants ranked all the (optional) categories.

A further limitation is that the young people’s survey was carried out only with students from one specialist school. These schools are uncommon and are not representative of the whole population of children in the UK who are affected in their education by having experienced trauma. ‘Children with a history of trauma’ is a challenging group to define and to seek out when researching a sensitive topic. Questions and priorities will vary among populations so it is important to try and get a large and varied sample base, which is something to be improved upon from the child aspect of this PPI. Future studies could explore alternate methods, such as involving a larger sample of parent/carer/teacher–child dyads, and providing an activity pack to generate questions together facilitated by a video call or face to face visit with a researcher either at home or school settings. Schools could be utilised to identify and support involvement of families, with the researcher being available at the school to meet parents and carers prior to starting the question generating process.

The unknown demographic information in the adult sample and the disparity between the number of questions in each of the themes in the adult stakeholder survey also meant that the opportunity to carry out statistical analysis to determine one question as the ultimate priority was limited. As well as this, an extensive research evidence base exists for many of the questions brought up in the prioritisation process. These were not removed in order to understand whether the PPI priorities aligned with the literature. To utilise these prioritised questions in future research, it remains to be established which questions would be novel contributions to the field. In the immediate future, knowledge dissemination efforts could be made, as detailed above.

Staff did draw attention to the fact that some questions proposed are regularly asked by students and they are given answers to these, highlighting the need for caution in excessive repetition or disseminating knowledge that is perceived to be commonplace, which may call into question the utility of such communications. It is therefore worth investing time into the child-facing prioritisation process to establish a common protocol which can be adapted to ensure the validity of the questions collected, perhaps by discussing face validity of all questions with a trusted adult, if the exercise is carried out in adult–child dyads in future. However, this may be inappropriate or invalidate the child’s voice if the adult feels it has been repeatedly answered or is not important. Indeed, when asked about improvements to be made to future children-facing processes, members of staff suggested that the practice was rewarding for students, but that they would have preferred to meet and carry out the two question collections with researchers instead of their familiar school staff (a step which we had avoided as familiarity of the facilitator was presumed to have been helpful in this process). As well as this, the format of the list could have been improved—the questions were originally presented in a list form, but to improve this method teachers suggested utilising “this or that” choices, presenting questions two at a time, perhaps even working with themes rather than specific questions. A balance between generating questions with independent adults, ensuring young people feel comfortable, and adults who are responsible for them being aware of the questions they consider important is needed. One option would be to carry out the exercise in parallel adult and children groups, then swap the questions so adults and children could have an immediate opportunity to review and reflect on questions posed at a group level. Limits on the number of questions to be put forward may be needed in light of the other feedback above.

### Recommendations for Future Research

A challenge to be considered in the future is how best to conceptualise questions proposed by the lay audience in a way as to make them researchable with a robust scientific method without losing the essence of what the proposers are asking. This is a challenge because many questions were very broad in nature, and to conduct a research study very precise decisions must be made about exactly what to measure, how and when to measure it, who specifically should participate in the research, and what precise question will be answered. The challenge of including children in research has also been noted by the James Lind Alliance, who are soon to release guidance on involving children in priority setting partnerships (*News and Publications | James Lind Alliance*, n.d.).

The fact that the questions that received most votes have existing answers suggests that many of the participating stakeholders were not aware of this knowledge base. Despite information being available for parents, (for example this fact sheet made by the children’s bureau (Parenting a Child Who Has Experienced Trauma, 2014)), a major theme was the challenge in dissemination outside of specialist educational provisions. This is highlighted by one of the priority questions: “*How can schools work in partnership with families?”* and was also a point raised by many in the focus group who experience the disparity between the training available to school practitioners and the knowledge readily available to parents. Because of this, further work is needed to identify which of the top priorities are true knowledge gaps and which have been inadequately communicated. Further research could also advise on how this knowledge gap can be best bridged. This considered, it is also a recommendation that any future research carried out in the field be collaborative, using multiple approaches and interested stakeholders to avoid seeing the same pattern repeated in the future: that of a gap in the public knowledge.

As well as this, the number of respondents to each of the adult stakeholder surveys suggests that more individuals were open to suggesting ideas than to ranking others’ ideas. The first survey was available for a longer time, however, the majority of responses occurred to both forms within the first few weeks. This could be due to the concept of submitting a question appearing more engaging, or the form for the second survey was not accessible enough, in which case steps could be taken to improve this in the future. Further research is required also to establish the priorities of students with histories of trauma who attend mainstream schools as opposed to specialist alternate provisions. Furthermore, insights from all children on the topic, whether or not they have experienced trauma themselves, could be valuable contributions, and offer a novel approach.

Despite some limitations, these prioritised research questions provide an opportunity to understand from a public instead of a researcher's perspective what the future priorities should be for education with children who have experienced trauma. These questions can now be taken forward to inform future dissemination, funding, research, and communications decisions.

## Conclusion

Through two research prioritisation processes in two different populations (made up of adults with lived or professional experience and children who have experienced trauma) we discovered that there is some overlap between perceived priorities and the topics that are currently well represented in the literature, namely how trauma-informed learning and care can be implemented in the mainstream schooling system. However, almost half of the respondents were parents or carers, which may highlight that the most prioritised issues were priorities held by parents more than teachers and school staff—a perspective which was also felt in the focus group. Children and young people’s questions largely revolved around reasonings and explanations, whereas questions from adults tended to focus on actions and facts. The children and young people also raised questions that some specialists in the focus group were surprised by, thus showing the importance of involving young people in research. Priorities from each group have been shown and it is intended that these will be taken into consideration by researchers and funders alike when proposing future research.

## Supplementary Information

Below is the link to the electronic supplementary material.

Supplementary file1 (DOCX 485 KB)

Supplementary file2 (DOCX 161 KB)

Supplementary file3 (DOCX 2431 KB)

Supplementary file4 (DOCX 141 KB)

Supplementary file5 (DOCX 25 KB)

Supplementary file6 (DOCX 43 KB)

Supplementary file7 (DOCX 26 KB)

## References

[CR1] *Adoption UK Charity*. (n.d.). Adoption UK charity. Retrieved 10 October 2022, from https://www.adoptionuk.org/. Accessed 09/02/2024.

[CR2] Alisic, E. (2012). Teachers’ perspectives on providing support to children after trauma: A qualitative study. *School Psychology Quarterly : The Official Journal of the Division of School Psychology, American Psychological Association,**27*, 51–59. 10.1037/a0028590 22582936 10.1037/a0028590

[CR3] Alisic, E., Zalta, A. K., van Wesel, F., Larsen, S. E., Hafstad, G. S., Hassanpour, K., & Smid, G. E. (2014). Rates of post-traumatic stress disorder in trauma-exposed children and adolescents: Meta-analysis. *The British Journal of Psychiatry,**204*(5), 335–340. 10.1192/bjp.bp.113.131227 24785767 10.1192/bjp.bp.113.131227

[CR4] Anda, R. F., Felitti, V. J., Bremner, J. D., Walker, J. D., Whitfield, C., Perry, B. D., Dube, S. R., & Giles, W. H. (2006). The enduring effects of abuse and related adverse experiences in childhood. *European Archives of Psychiatry and Clinical Neuroscience,**256*(3), 174–186. 10.1007/s00406-005-0624-4 16311898 10.1007/s00406-005-0624-4 PMC3232061

[CR5] Avery, J. C., Morris, H., Galvin, E., Misso, M., Savaglio, M., & Skouteris, H. (2021). Systematic review of school-wide trauma-informed approaches. *Journal of Child & Adolescent Trauma,**14*(3), 381–397. 10.1007/s40653-020-00321-1 34471456 10.1007/s40653-020-00321-1 PMC8357891

[CR6] Bremner, J. D. (2006). Traumatic stress: Effects on the brain. *Dialogues in Clinical Neuroscience,**8*(4), 445–461. 17290802 10.31887/DCNS.2006.8.4/jbremner PMC3181836

[CR7] Brett, J., Staniszewska, S., Mockford, C., Herron-Marx, S., Hughes, J., Tysall, C., & Suleman, R. (2014). A systematic review of the impact of patient and public involvement on service users, researchers and communities. *The Patient - Patient-Centered Outcomes Research,**7*(4), 387–395. 10.1007/s40271-014-0065-0 25034612 10.1007/s40271-014-0065-0

[CR8] Bronfenbrenner, U. (1974). Developmental research, public policy, and the ecology of childhood. *Child Development,**45*(1), 1–5. 10.2307/1127743

[CR9] Bruce, A. D. F. and S. E. (2010, June 7). *Impact of exposure to community violence on violent behavior and emotional distress among urban adolescents | EndNote Click*. https://click.endnote.com/viewer?doi=10.1207%2Fs15374424jccp2601_1&token=WzM2Mzc4MjYsIjEwLjEyMDcvczE1Mzc0NDI0amNjcDI2MDFfMSJd.uNQBH9KH8ffWogKFcs80qGBtTQw. Accessed 09/02/2024. 10.1207/s15374424jccp2601_1 9118172

[CR10] Brunzell, T., Stokes, H., & Waters, L. (2019). Shifting teacher practice in trauma-affected classrooms: Practice pedagogy strategies within a trauma-informed positive education model. *School Mental Health,**11*(3), 600–614. 10.1007/s12310-018-09308-8

[CR12] Chambers Concise Dictionary. (2004). Available at: https://chambers.co.uk/search/?query=trauma&title=21st. Accessed 15 May 2025.

[CR13] Cheong, E. V., Sinnott, C., Dahly, D., & Kearney, P. M. (2017). Adverse childhood experiences (ACEs) and later-life depression: Perceived social support as a potential protective factor. *British Medical Journal Open,**7*(9), e013228. 10.1136/bmjopen-2016-013228 10.1136/bmjopen-2016-013228 PMC5588961 28864684

[CR14] Child Welfare Information Gateway. (2011). *Supporting brain development in traumatized children and youth: (552932013-001)* [dataset]. American Psychological Association. 10.1037/e552932013-001

[CR15] Desforges, C., & Abouchaar, A. (2003). *The impact of parental involvement, parental support and family education on pupil achievements and adjustment: A literature review*. DfES.

[CR16] Douglas Bremner, J., Randall, P., Scott, T. M., Capelli, S., Delaney, R., McCarthy, G., & Charney, D. S. (1995). Deficits in short-term memory in adult survivors of childhood abuse. *Psychiatry Research,**59*(1), 97–107. 10.1016/0165-1781(95)02800-5 8771224 10.1016/0165-1781(95)02800-5

[CR17] Felitti, V. J., Anda, R. F., Nordenberg, D., Williamson, D. F., Spitz, A. M., Edwards, V., Koss, M. P., & Marks, J. S. (1998). Relationship of childhood abuse and household dysfunction to many of the leading causes of death in adults: The adverse childhood experiences (ACE) study. *American Journal of Preventive Medicine,**14*(4), 245–258. 10.1016/S0749-3797(98)00017-8 9635069 10.1016/s0749-3797(98)00017-8

[CR18] Ferguson, D. (2019, November 10). Record levels of stress ‘put teachers at breaking point’. *The observer*. https://www.theguardian.com/education/2019/nov/10/stressed-teachers-at-breaking-point-says-report

[CR19] Fitzpatrick, K. M., & Boldizar, J. P. (1993). The prevalence and consequences of exposure to violence among African-American youth. *Journal of the American Academy of Child & Adolescent Psychiatry,**32*(2), 424–430. 10.1097/00004583-199303000-00026 8444774 10.1097/00004583-199303000-00026

[CR20] Forbes, C., Morley, N., Liabo, K., Bjornstad, G., Boult, H., Ahmed, S., Ciesla, K., Vafai, Y., Bridges, S., Logan, S., & Berry, V. (2022). Prioritising child health and maternity evidence-based interventions or service models: A stakeholder-driven process. *BMC Health Services Research,**22*(1), 764. 10.1186/s12913-022-08110-2 35689231 10.1186/s12913-022-08110-2 PMC9186012

[CR21] Formula, O. (n.d.). *Touchbase*. Touchbase. Retrieved 10 October 2022, from https://touchbase.org.uk/. Accessed 09/02/2024.

[CR22] Grotz, J., Ledgard, M., spsampsps Poland, F. (2020). *Patient and public involvement in health and social care research: An introduction to theory and practice*. Springer International Publishing. 10.1007/978-3-030-55289-3

[CR23] Horobin, D. A., Prof Deborah Hall Dr Adele Horobin is, Public, & programme. (2018, March 2). *No research about me without me – Why researchers should welcome the patient’s voice*. On medicine. https://blogs.biomedcentral.com/on-medicine/2018/03/02/no-research-about-me-without-me-researchers-welcome-patients-voice/. Accessed 09/02/2024.

[CR24] Hurt, H., Malmud, E., Brodsky, N. L., & Giannetta, J. (2001). Exposure to violence: Psychological and academic correlates in child witnesses. *Archives of Pediatrics & Adolescent Medicine,**155*(12), 1351–1356. 10.1001/archpedi.155.12.1351 11732955 10.1001/archpedi.155.12.1351

[CR25] Martinez, P., & Richters, J. E. (1993). The Nimh community violence project: II. Children’s distress symptoms associated with violence exposure. *Psychiatry,**56*(1), 22–35. 10.1080/00332747.1993.11024618 8488209 10.1080/00332747.1993.11024618

[CR26] Merrick, M. T., Ford, D. C., Ports, K. A., et al. (2019). Vital signs: Estimated proportion of adult health problems attributable to adverse childhood experiences and implications for prevention — 25 states, 2015–2017. *MMWR. Morbidity and Mortality Weekly Report,**68*, 999–1005. 10.15585/mmwr.mm6844e1 31697656 10.15585/mmwr.mm6844e1 PMC6837472

[CR27] *Microsoft Forms*. (n.d.). Retrieved 24 October 2023, from https://forms.office.com/Pages/DesignPageV2.aspx. Accessed 09/02/2024.

[CR28] Murphy, J. M., Guzmán, J., McCarthy, A., Squicciarini, A. M., George, M., Canenguez, K., Dunn, E. C., Baer, L., Simonsohn, A., Smoller, J. W., & Jellinek, M. (2015). Mental health predicts better academic outcomes: A longitudinal study of elementary school students in Chile. *Child Psychiatry and Human Development,**46*(2), 245–256. 10.1007/s10578-014-0464-4 24771270 10.1007/s10578-014-0464-4 PMC4443903

[CR29] *News and publications | James Lind Alliance*. (n.d.). Retrieved 24 October 2023, from https://www.jla.nihr.ac.uk/news/developing-guidance-for-involving-children-and-young-people-in-psps/34680. Accessed 09/02/2024.

[CR30] *NVivo*. (n.d.). [Computer software]. Lumivero. www.lumivero.com. Accessed 09/02/2024.

[CR31] *Online surveys*. (n.d.). Retrieved 24 October 2023, from https://www.onlinesurveys.ac.uk/. Accessed 09/02/2024.

[CR32] Parenting a Child Who has Experienced Trauma. (2014). *Child welfare information gateway*. Available at: https://www.childwelfare.gov/resources/parenting-child-who-has-experienced-trauma/. Accessed 15 May 2025.

[CR33] Perfect, M. M., Turley, M. R., Carlson, J. S., Yohanna, J., & Saint Gilles, M. P. (2016). School-related outcomes of traumatic event exposure and traumatic stress symptoms in students: A systematic review of research from 1990 to 2015. *School Mental Health,**8*(1), 7–43. 10.1007/s12310-016-9175-2

[CR34] *Plymouth Trauma Informed Network – Plymouth Octopus*. (n.d.). Retrieved 10 October 2022, from https://www.plymouthoctopus.org/network/plymouth-trauma-informed-network/. Accessed 09/02/2024.

[CR35] Purtle, J. (2020). Systematic review of evaluations of trauma-informed organizational interventions that include staff trainings. *Trauma, Violence, & Abuse,**21*(4), 725–740. 10.1177/1524838018791304 10.1177/1524838018791304 30079827

[CR36] Souers, K., & Hall, P. (2016). *Fostering resilient learners: Strategies for creating a trauma-sensitive classroom*. ASCD.

[CR37] Stein, B. D., Jaycox, L. H., Kataoka, S. H., Wong, M., Tu, W., Elliott, M. N., & Fink, A. (2003). A mental health intervention for schoolchildren exposed to violence. A randomized controlled trial. *JAMA,**290*(5), 603–611. 10.1001/jama.290.5.603 12902363 10.1001/jama.290.5.603

[CR38] Tikellis, G., Tong, A., Lee, J. Y. T., Corte, T. J., Hey-Cunningham, A. J., Bartlett, M., Crawford, T., Glaspole, I., Price, J., Maloney, J., & Holland, A. E. (2021). Top 10 research priorities for people living with pulmonary fibrosis, their caregivers, healthcare professionals and researchers. *Thorax,**76*(6), 575–581. 10.1136/thoraxjnl-2020-215731 33277429 10.1136/thoraxjnl-2020-215731

[CR39] *Trauma sensitive practice with children in** care*. (2014, August 13). Iriss. https://www.iriss.org.uk/resources/insights/trauma-sensitive-practice-children-care. Accessed 09/02/2024.

