# Supplementary material for: What Next for Trauma-Informed Education Research? A Research Prioritisation Exercise with Young People as Informants
Source: J Child Adolesc Trauma. 2025 May 23;18(3):803–13. doi: 10.1007/s40653-025-00711-3 (PMC12433405; doi:10.1007/s40653-025-00711-3)

**What next for trauma-informed education research? A research prioritisation exercise with young people as informants.**

**Staff Info Sheet – Question Collecting**

Our overall goal: **hear the voices of children with complex backgrounds and find out what they want to know about their education.**

This is **part of a larger study**, but information from young people is **only being collected at [xxx]**

These are some examples of questions we have **already been asked** as part of the adult section of the study:

- Do forest schools result in better engagement with education for children with trauma?
- Are children in education receiving a good balance of physical education, drama and creative arts to help them manage their individual trauma and time given to listen to their individual needs?
- How do schools better meet the needs of children with a trauma history where there is no specific diagnosis?

We need your help: You know the students at your school incredibly well. We want you to help them **have their voices heard.**

To do this we need your assistance **collecting questions** from students, during 1:1 sessions in most cases, but this may also occur during PSHE time if the class will interact with that positively.

We appreciate that what we are asking of the young people is a challenging task, and we need your help to nudge them in the right direction.

You may want to start off with an **example of what asking questions about something looks like**:

**Class names: Rivers**

Where is the river that is my class name?

How deep is the river?

How long is the river?

What makes the river bend?

**Blu the dog:**

Why does Blu wag his tail when he is happy?

What is Blu’s favourite treat?

Is Blu a big or a small dog compared to other dogs of his type?

**Last term theme: space**

How do astronauts breathe in space?

How long does it take to get to the moon?


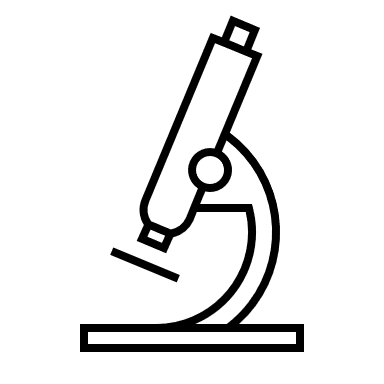

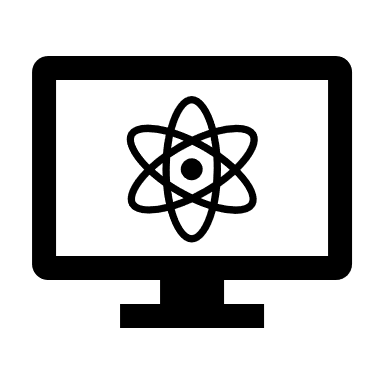

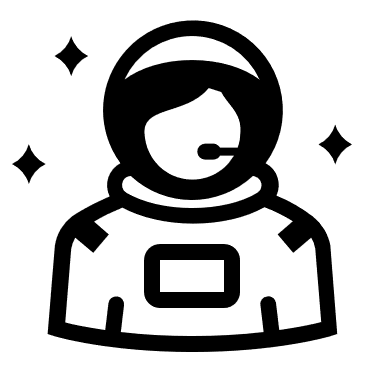

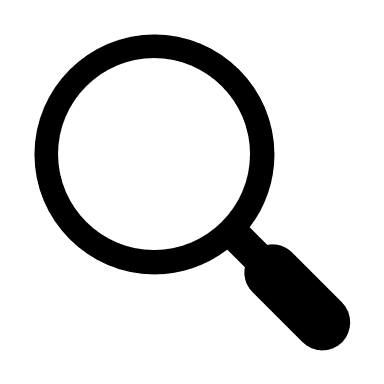

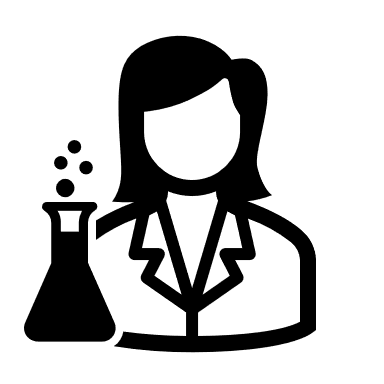
These are things that **we may not know** but **scientists will be able to investigate!**

You might then encourage the student to talk about questions that they have about **themselves**, their **education or school experiences**, or their **backgrounds.**

Questions about these things may look like:

- Why do I feel more calm when I am in the immersion room?
- Why do I _________ when ________ happens (eg why do I get stressed when the classroom is noisy)
- How does my ________ affect my ________ (eg how does my sleep during the night effect my mood the next day)
- Why do I remember more of the work I do in forest school?
- Why do I like it more at this school than my previous school?
- Why do different people react differently to things?
- Does everyone at this school like the immersion room?

**Note: These are only examples and can include anything about education of children with complex life stories.**

**These can take any form and do not have to be questions. They can just be ideas, phrases, or general areas, but it would be helpful to have some background information about the conversation you have had up to that point, which will help us to phrase it into a question.**

It is important that the young people understand that, although we will circulate the complete list of questions at the end of the study, their question may not appear on this list as it may have already been answered, and their question may not be picked as one of the top questions.

We will be carrying out data collection until the **11^th^ of March** at which point we will need to compile all of our questions to start forming our second list.

**To submit student questions either:**

- **Write on paper** and give to [xxx] (note name of class and class teacher on paper) OR
- Follow **QR code** OR
- **Follow link:** <https://forms.office.com/r/ndUaPc33iW>


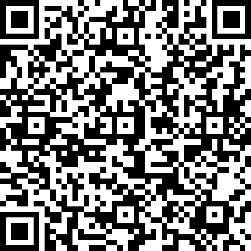


**
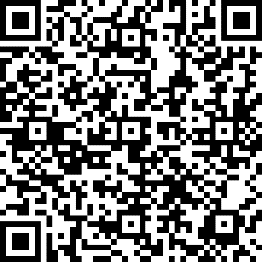
**

**To submit staff / parent/ guardian questions**

If you would like to **submit your own questions** to help us with this exercise, please follow this link: <https://forms.office.com/r/bVYA2wPBK2> or scan the QR code.

**Finally, thanks for all of your help! We couldn’t do this without you and we’re really excited to be working with you and the students at [xxx]**

**If you have any questions, please email [xxx]**

**Poster**


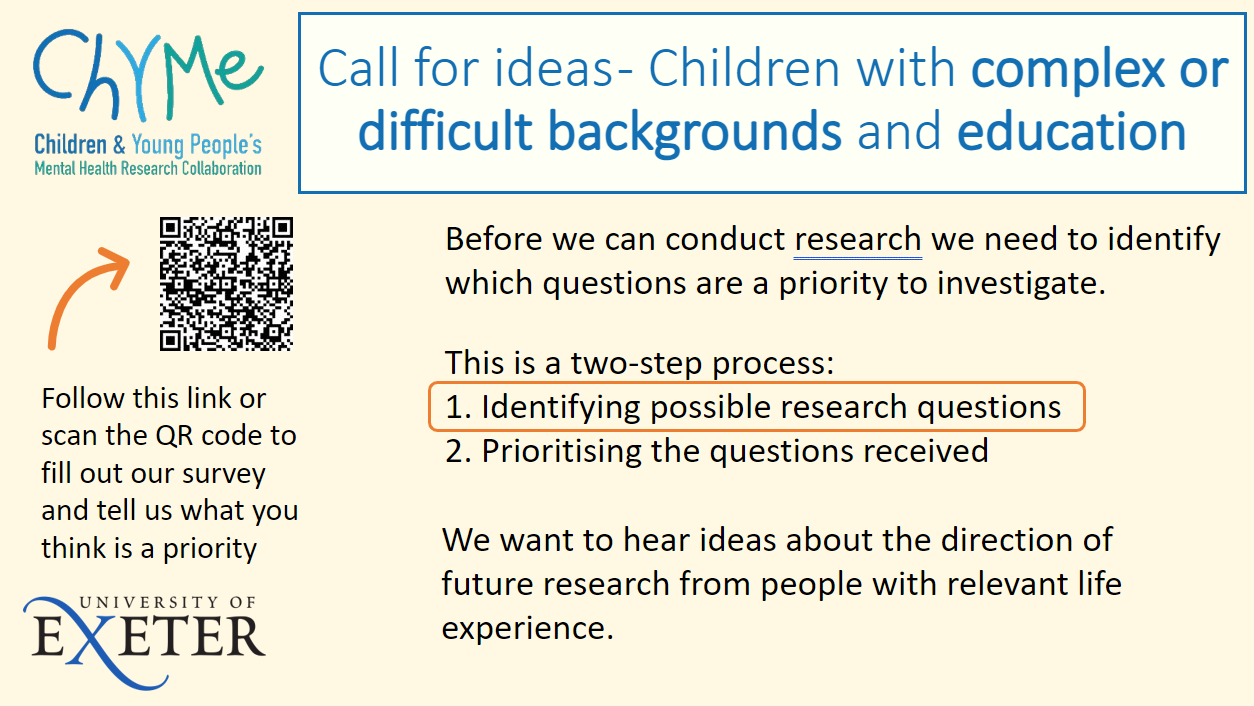


**Breakdown of the process of research prioritisation**
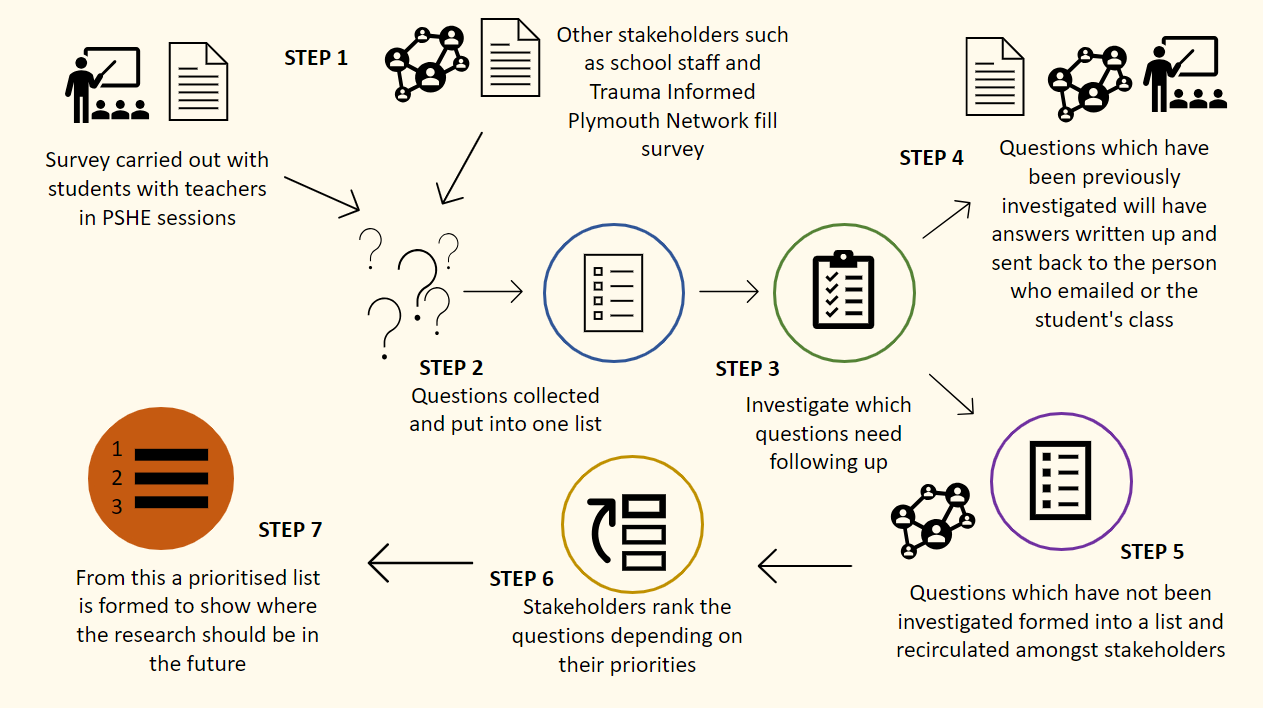

Supplement: Supplementary file 1 — Supplementary file1 (DOCX 485 KB) [file 40653_2025_711_MOESM1_ESM.docx]
