# Supplementary material for: What Next for Trauma-Informed Education Research? A Research Prioritisation Exercise with Young People as Informants
Source: J Child Adolesc Trauma. 2025 May 23;18(3):803–13. doi: 10.1007/s40653-025-00711-3 (PMC12433405; doi:10.1007/s40653-025-00711-3)

**What next for trauma-informed education research? A research prioritisation exercise with young people as informants.**


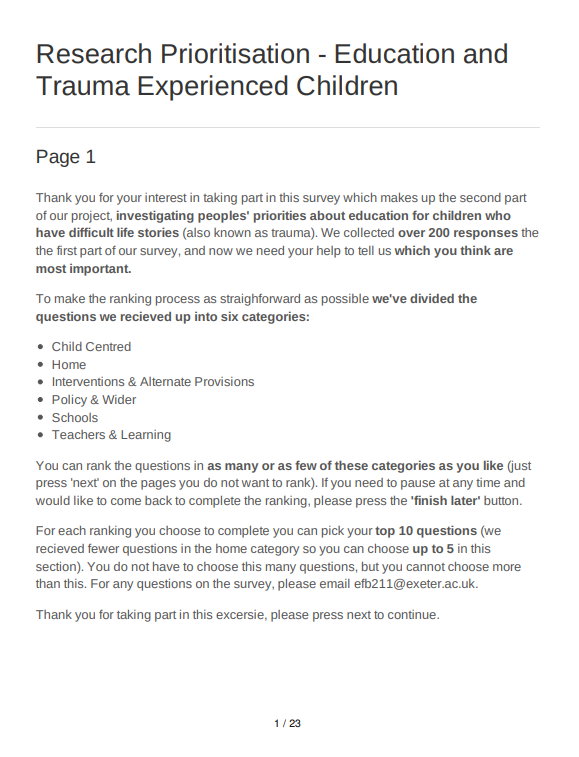


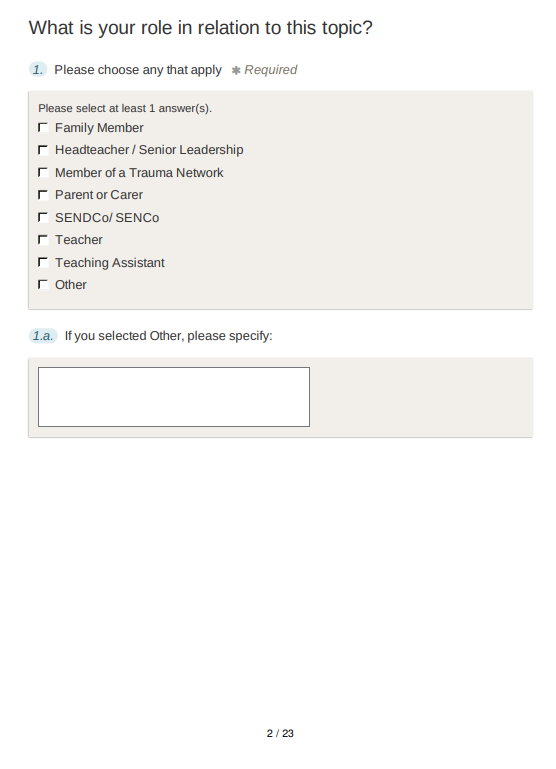


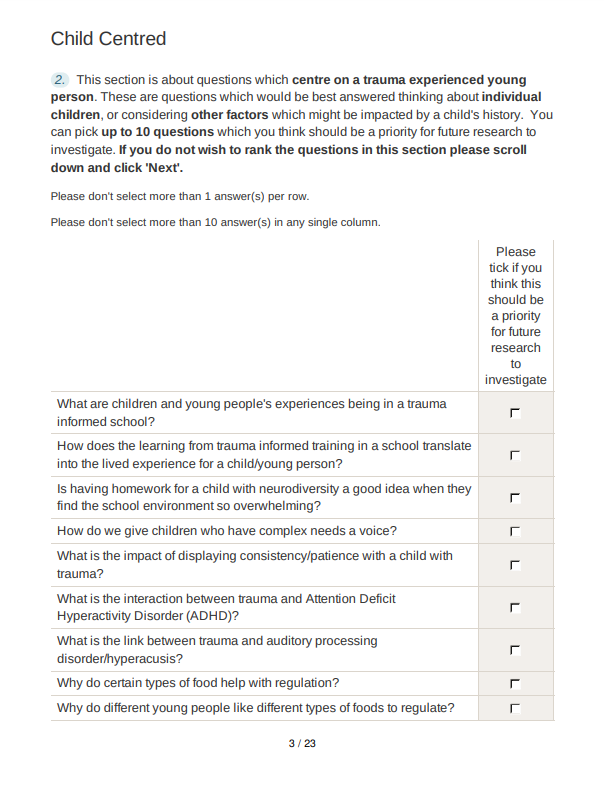


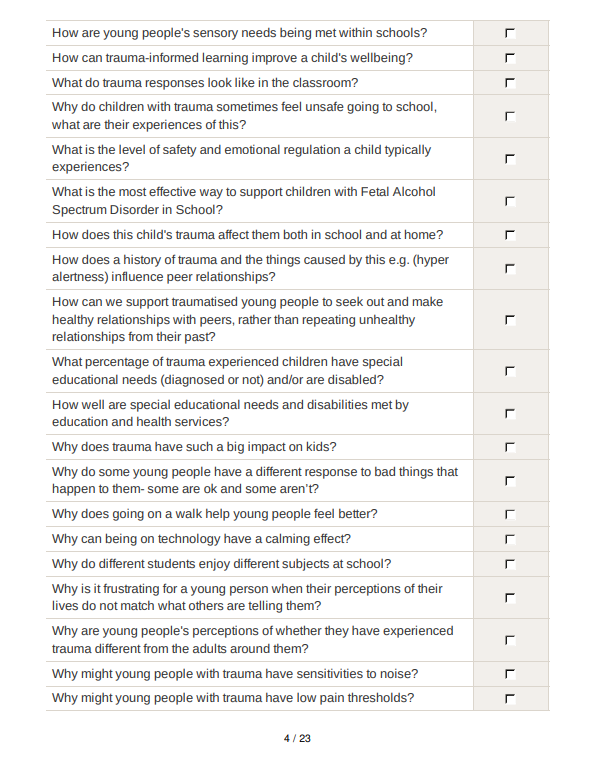


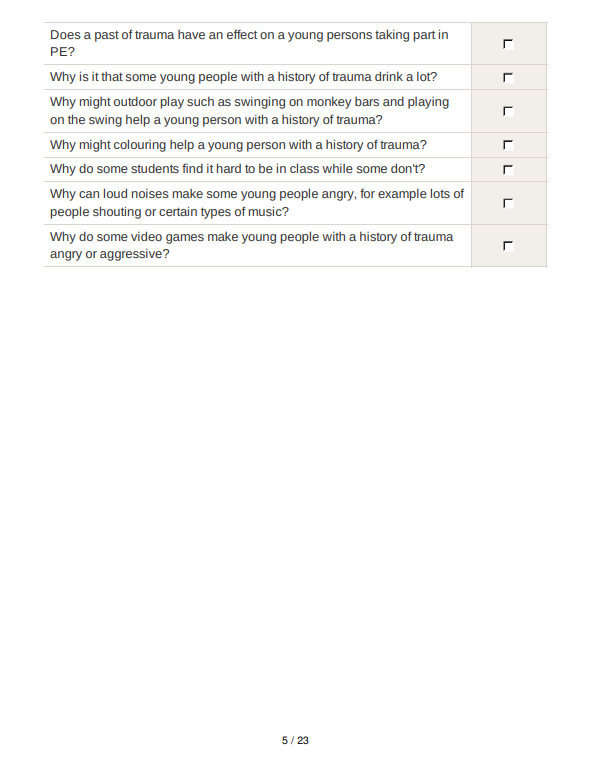


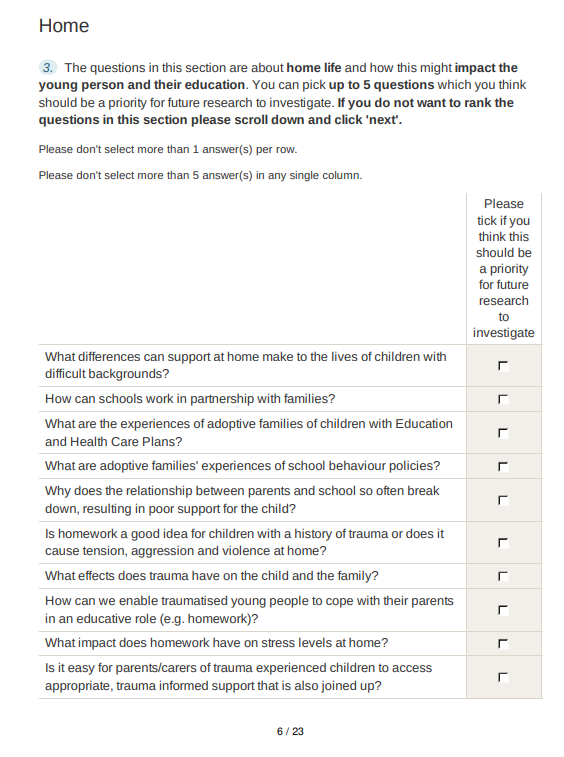


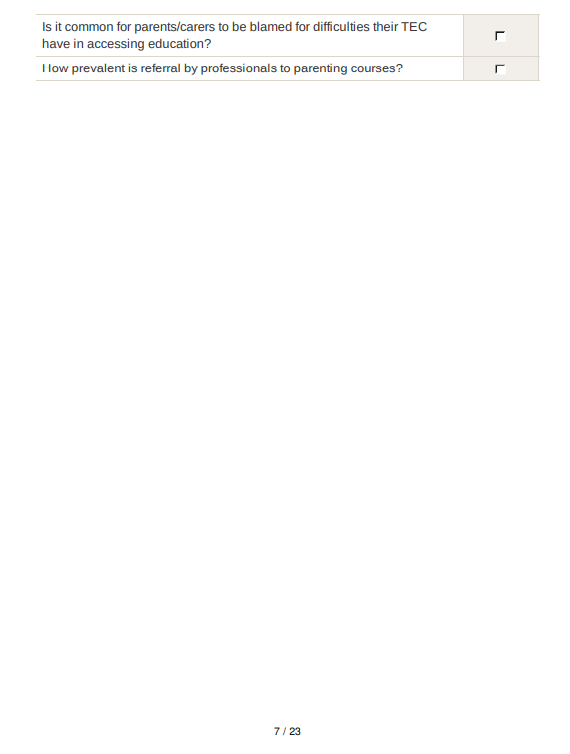

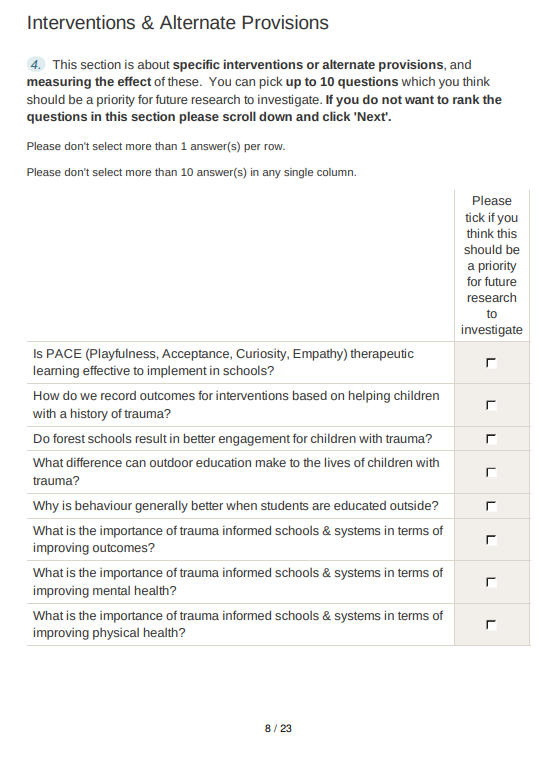

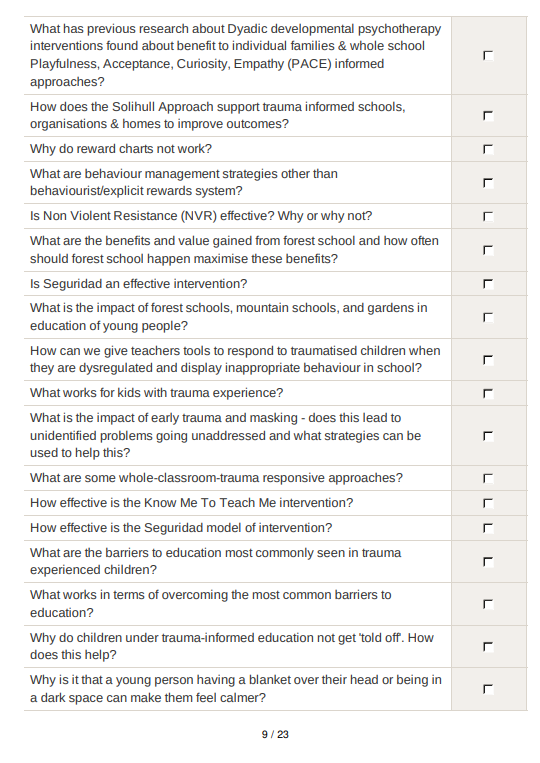

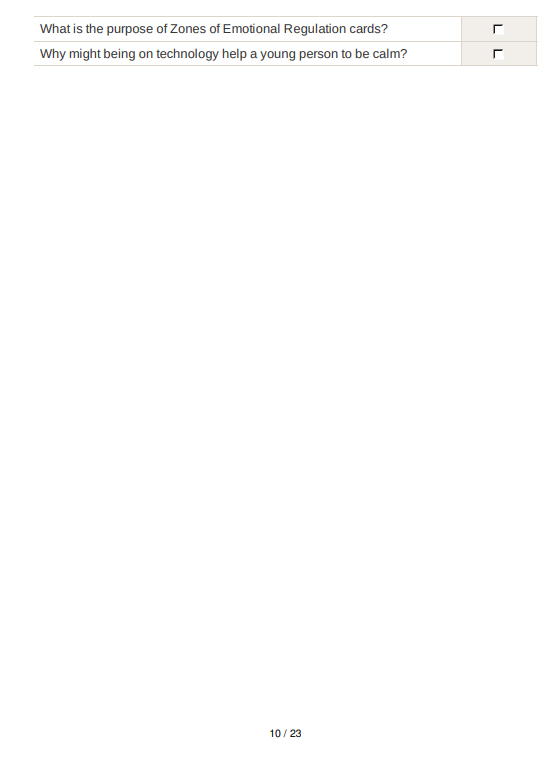


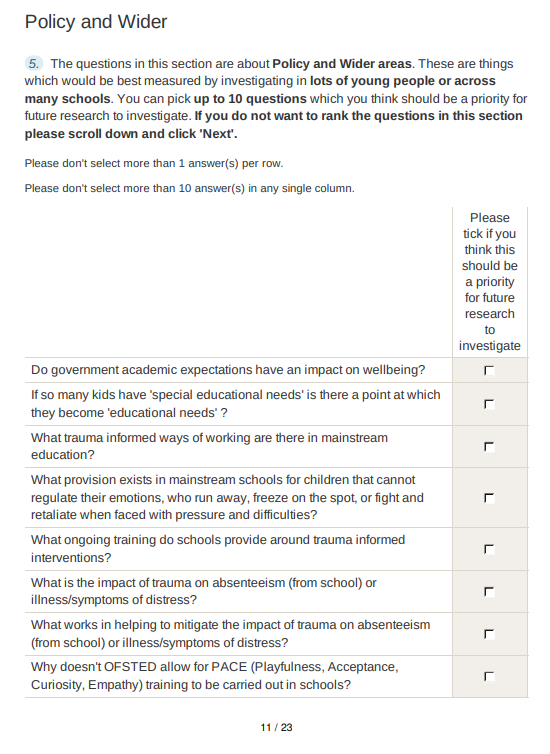


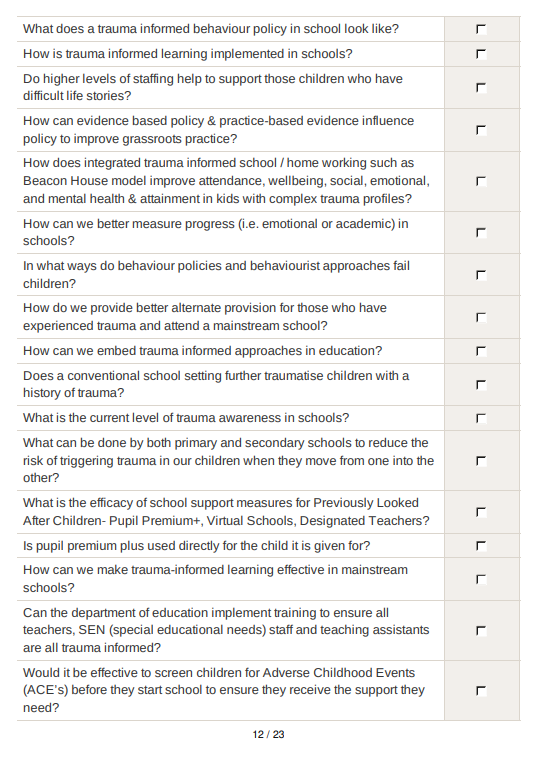

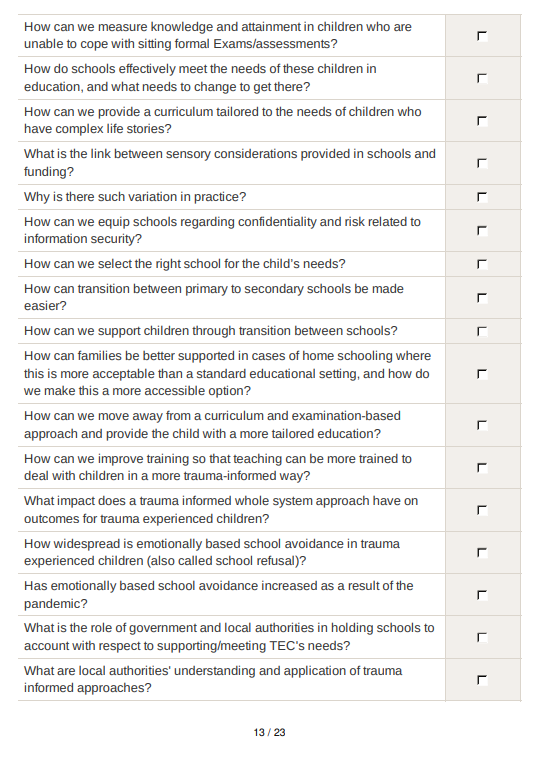


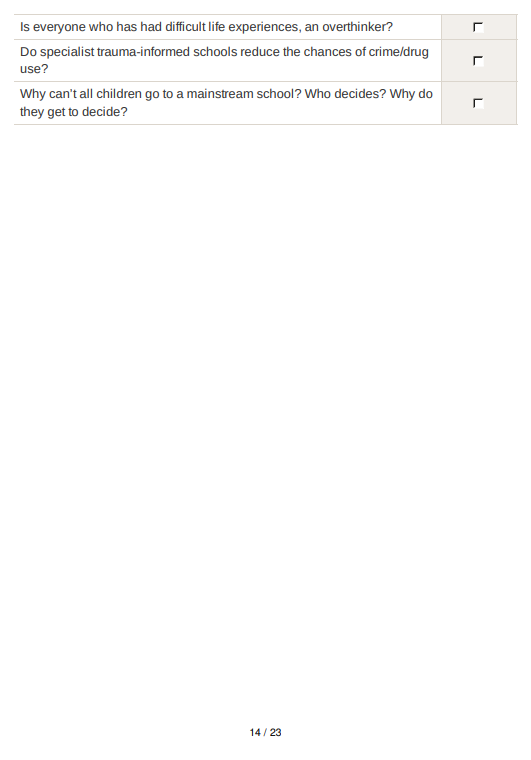


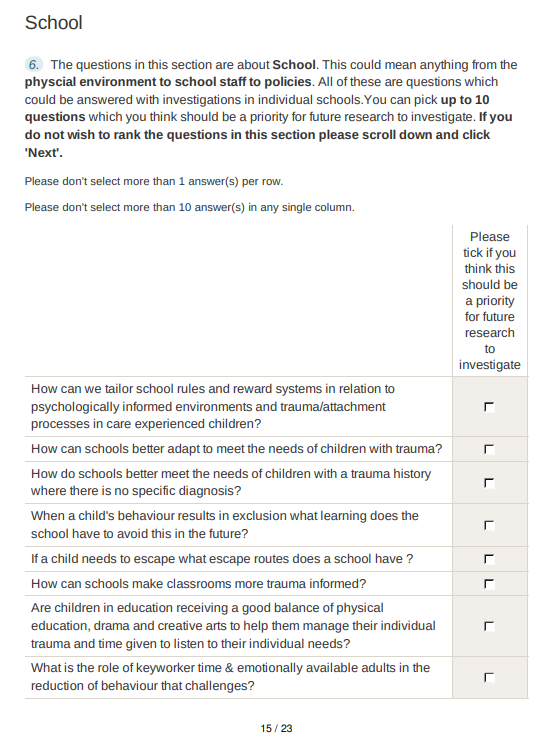


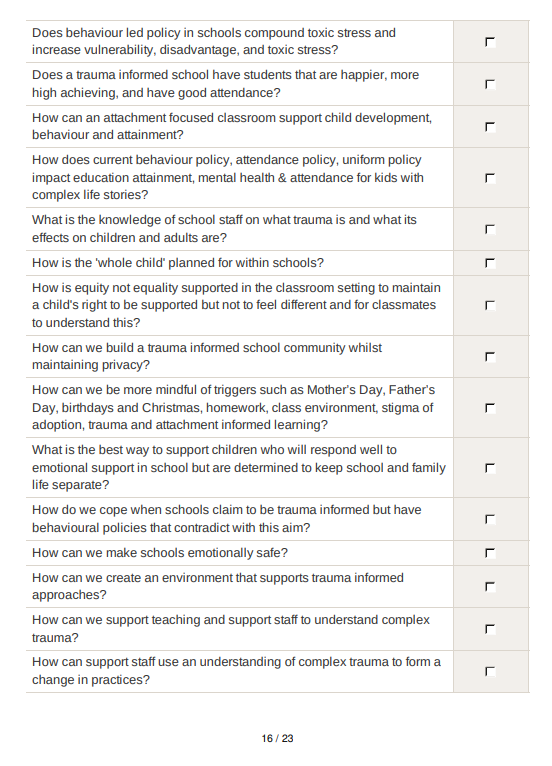


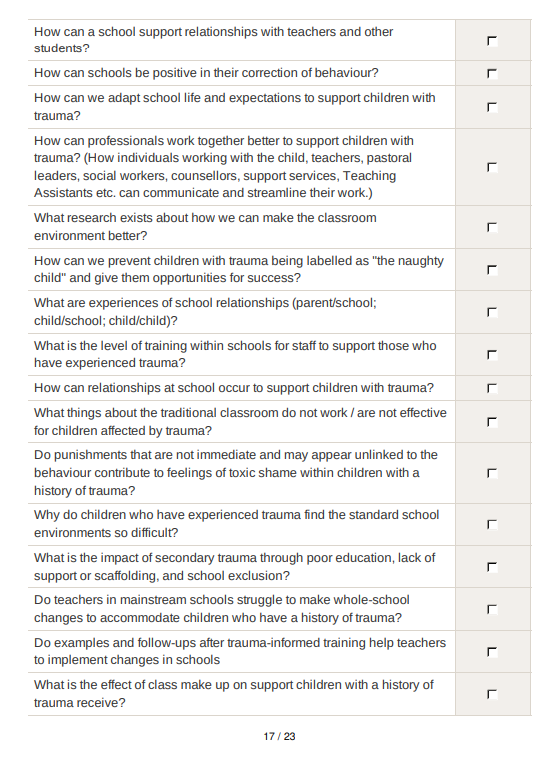


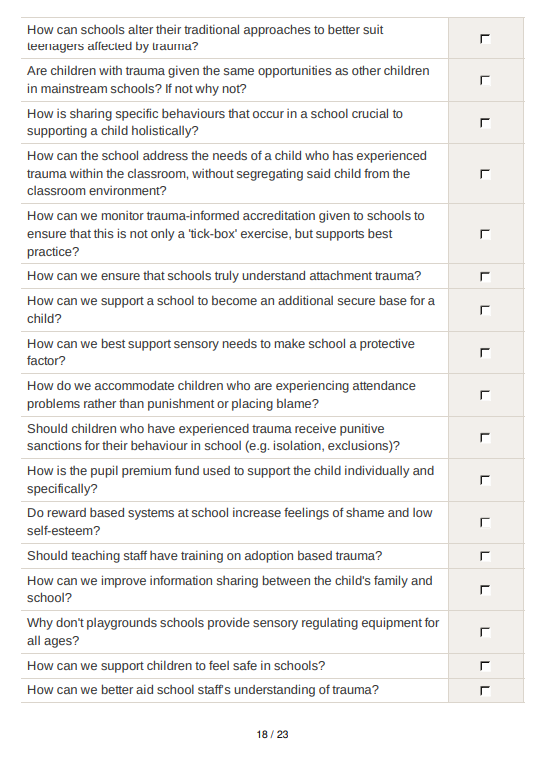


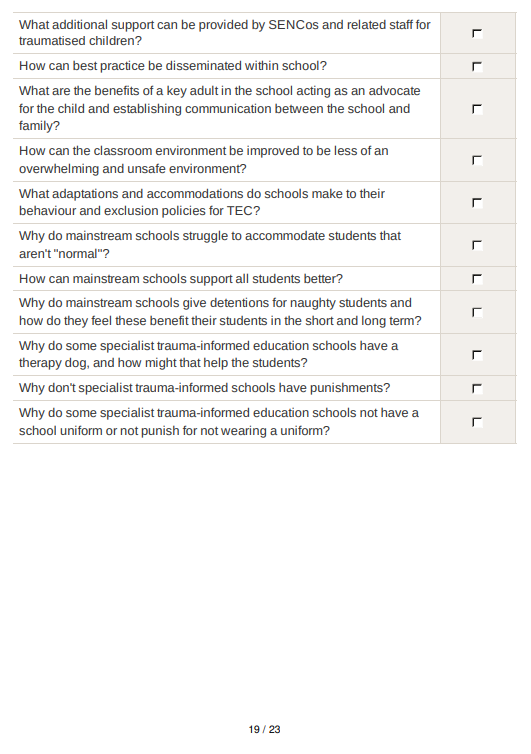


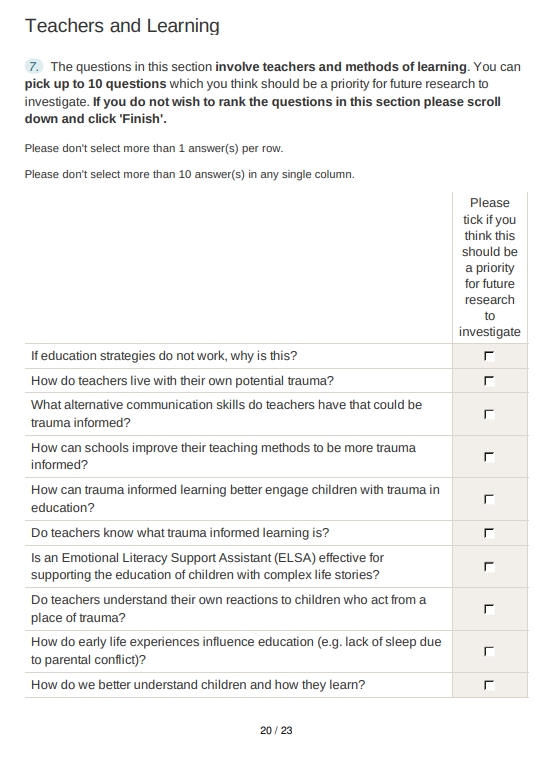


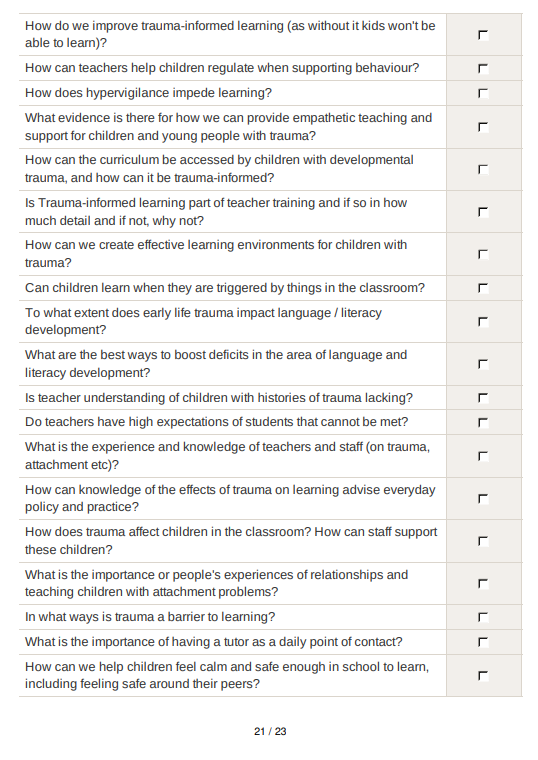


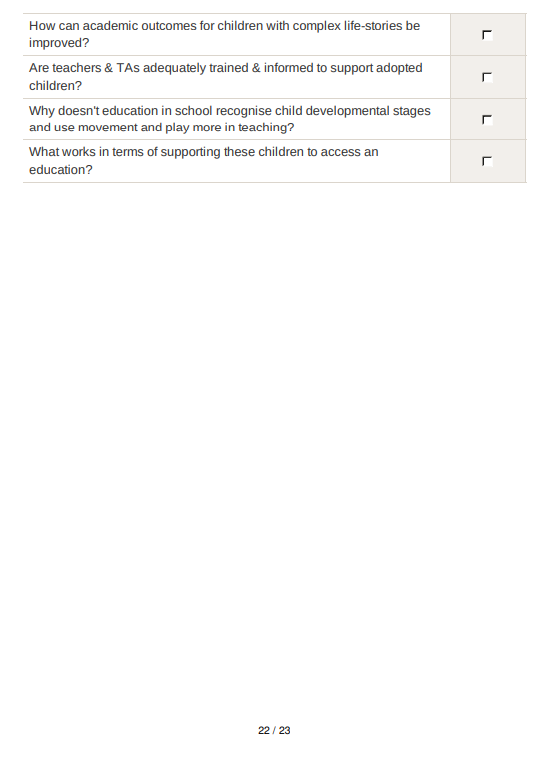


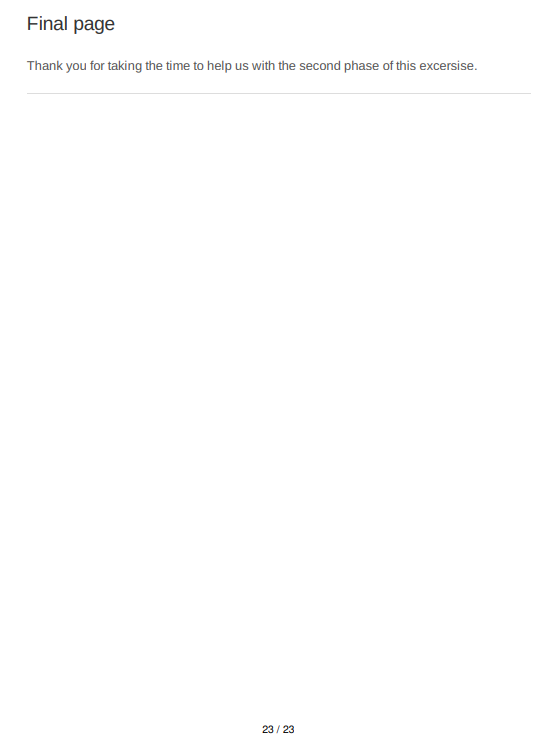

Supplement: Supplementary file 3 — Supplementary file3 (DOCX 2431 KB) [file 40653_2025_711_MOESM3_ESM.docx]
