# Supplementary material for: What Next for Trauma-Informed Education Research? A Research Prioritisation Exercise with Young People as Informants
Source: J Child Adolesc Trauma. 2025 May 23;18(3):803–13. doi: 10.1007/s40653-025-00711-3 (PMC12433405; doi:10.1007/s40653-025-00711-3)
Supplement: Supplementary file 5 — Supplementary file5 (DOCX 25 KB) [file 40653_2025_711_MOESM5_ESM.docx]

**What next for trauma-informed education research? A research prioritisation exercise with young people as informants.**

**Hello! We would like to know which of these questions you think are most important to do research on.**

**Some of the questions might sound familiar – this is because they might have been asked by you or someone at your school.**

**Please put a tick next to THREE QUESTIONS that you think are the most important or urgent for research to answer.**

| - Why does trauma have such a big impact on kids? | II |
| --- | --- |
| - Why do some kids have a different response to bad things that happen to them: some are ok and some aren’t? | II |
| - Why do I feel better when I go for a walk? | II |
| - Is everyone who’s had difficult life experiences, an overthinker? | III |
| - Do special schools like ours reduce the chances of crime/drug use? | II |
| - Why don’t mainstream schools understand people that aren’t “normal”? | II |
| - Why don’t we get consequences/told off – how does this help us? | I |
| - Why does being on technology help us be calm? | III |
| - How can mainstream schools support all students better? |  |
| - Why do different people enjoy different subjects? | I |
| - Why do I get so mad when people assume I’ve been through trauma when I haven’t? | I |
| - Why do people say I’ve experienced trauma when I don’t think I have? | I |
| - Why am I sensitive to noise? | I |
| - Why do I have a low pain threshold? |  |
| - Why do mainstream schools give detentions for naughty students and how do they feel these benefit their students in the short and long term? | I |
| - Could it be due to my background and trauma that I never want to participate in sports at school? | I |
| - Why is it that a blanket over my head or a dark space help me to calm down when I feel fizzy? |  |
| - Why do I always drink a lot? |  |
| - Why do we have a therapy dog and how does he help us? |  |
| - Why do we use Zones Cards? (Zones of Emotional Regulation) |  |
| - Why does being on technology help me be calm? |  |
| - Why does swinging on the monkey bars and playing on the swing help me? |  |
| - Why does colouring help me? |  |
| - Why don’t we have punishments? | I |
| - Why do some people find it harder to be in a classroom than others? |  |
| - Why don’t we get in trouble for not wearing school uniform? | I |

**Thank you for your help in our research!**
