# Supplementary material for: What Next for Trauma-Informed Education Research? A Research Prioritisation Exercise with Young People as Informants
Source: J Child Adolesc Trauma. 2025 May 23;18(3):803–13. doi: 10.1007/s40653-025-00711-3 (PMC12433405; doi:10.1007/s40653-025-00711-3)
Supplement: Supplementary file 6 — Supplementary file6 (DOCX 43 KB) [file 40653_2025_711_MOESM6_ESM.docx]

**What next for trauma-informed education research? A research prioritisation exercise with young people as informants.**

Top 5 Questions in Each Category

**Research Prioritisation Summary –**
**Education for Children with Trauma / Complex Life Stories**

**This document contains the top 5 questions from each category of the adult stakeholder questionnaire. The top student questions are not ranked due to many questions having the same number of votes.**

**If you would like further information please contact: [xxx]**

**Child-Centred**

1. **What do trauma responses look like in the classroom?**
2. How can trauma-informed learning improve a child's wellbeing?
3. How does the learning from trauma informed training in a school translate into the lived experience for a child/young person?
4. How do we give children who have complex needs a voice?
5. How can we support traumatised young people to seek out and make healthy relationships with peers, rather than repeating unhealthy relationships from their past?

**Home**

- **How can schools work in partnership with families?**
- What are adoptive families' experiences of school behaviour policies?
- Is it easy for parents/carers of trauma experienced children to access appropriate, trauma informed support that is also joined up?
- What are the experiences of adoptive families of children with Education and Health Care Plans?
- What effects does trauma have on the child and the family?

**Interventions & Alternate Provisions**

1. **How can we give teachers tools to respond to traumatised children when they are dysregulated and display inappropriate behaviour in school?**
2. What is the importance of trauma informed schools & systems in terms of improving outcomes?
3. What is the importance of trauma informed schools & systems in terms of improving mental health?
4. Is PACE (Playfulness, Acceptance, Curiosity, Empathy) therapeutic learning effective to implement in schools?
5. What difference can outdoor education make to the lives of children with trauma?

**Policy & Wider**

1. **What trauma informed ways of working are there in mainstream education?**
2. What does a trauma informed behaviour policy in school look like?
3. How can we embed trauma informed approaches in education?
4. How is trauma informed learning implemented in schools?
5. How does integrated trauma informed school / home working such as Beacon House model improve attendance, wellbeing, social, emotional, and mental health & attainment in kids with complex trauma profiles?

**School**

1. **How do schools better meet the needs of children with a trauma history where there is no specific diagnosis?**
2. How can schools better adapt to meet the needs of children with trauma?
3. How can we tailor school rules and reward systems in relation to psychologically informed environments and trauma/attachment processes in care experienced children?
4. How can schools make classrooms more trauma informed?
5. How can professionals work together better to support children with trauma? (How individuals working with the child, teachers, pastoral leaders, social workers, counsellors, support services, Teaching Assistants etc can communicate and streamline their work)

**Teachers and Learning**

1. **How can schools improve their teaching methods to be more trauma informed?**
2. Do teachers understand their own reactions to children who act from a place of trauma?
3. How does hypervigilance impede learning?
4. How can trauma informed learning better engage children with trauma in education?
5. Are teachers & TAs adequately trained & informed to support adopted children?

All Questions

**Research Prioritisation Summary –**
**Education for Children with Trauma / Complex Life Stories**

**This document lists all of the questions that were included in the student survey and the adult survey. Student questions were adapted and included in the adult surveys in order to compare adult and child priorities.**

**If you would like further information please contact: [xxx]**

Student Questions

Why does trauma have such a big impact on kids?

Why do some kids have a different response to bad things that happen to the: some are ok and some aren’t?

Why do I feel better when I go for a walk?

Is everyone who’s had difficult life experiences, an overthinker?

Do special schools like ours reduce the chances of crime/drug use?

Why don’t mainstream schools understand people that aren’t “normal”?

Why don’t we get consequences/told off – how does this help us?

Why does being on technology help us be calm?

How can mainstream schools support all students better?

Why do I get so mad when people assume I’ve been through trauma when I haven’t?

Why do I get so mad when people assume I’ve been through trauma when I haven’t?

Why do people say I’ve experienced trauma when I don’t think I have?

Why am I sensitive to noise?

Why do I have a low pain threshold?

Why do mainstream schools give detentions for naughty students and how do they feel these benefit their students in the short and long term?

Could it be due to my background and trauma that I never want to participate in sports at school?

Why is it that a blanket over my head or a dark space help me to calm down when I feel fizzy?

Why do I always drink a lot?

Why do we have a therapy dog and how does he help us?

Why do we use Zones Cards? (Zones of Emotional Regulation)

Why does swinging on the monkey bars and playing on the swing help me?

Why does colouring help me?

Why don’t we have punishments?

Why do some people find it harder to be in a classroom than others?

Why don’t we get in trouble for not wearing school uniform?

Adult Stakeholder Questions (by theme)

**Child-centred Questions**

What are children and young people's experiences being in a trauma informed school?

How does the learning from trauma informed training in a school translate into the lived experience for a child/young person?

Is having homework for a child with neurodiversity a good idea when they find the school environment so overwhelming?

How do we give children who have complex needs a voice?

What is the impact of displaying consistency/patience with a child with trauma?

What is the interaction between trauma and Attention Deficit Hyperactivity Disorder (ADHD)?

What is the link between trauma and auditory processing disorder/hyperacusis?

Why do certain types of food help with regulation?

Why do different young people like different types of foods to regulate?

How are young people's sensory needs being met within schools?

How can trauma-informed learning improve a child's wellbeing?

What do trauma responses look like in the classroom?

Why do children with trauma sometimes feel unsafe going to school, what are their experiences of this?

What is the level of safety and emotional regulation a child typically experiences?

What is the most effective way to support children with Fetal Alcohol Spectrum Disorder in School?

How does this child's trauma affect them both in school and at home?

How does a history of trauma and the things caused by this e.g. (hyper alertness) influence peer relationships?

How can we support traumatised young people to seek out and make healthy relationships with peers, rather than repeating unhealthy relationships from their past?

What percentage of trauma experienced children have special educational needs (diagnosed or not) and/or are disabled?

How well are special educational needs and disabilities met by education and health services?

Why does trauma have such a big impact on kids?

Why do some young people have a different response to bad things that happen to them- some are ok and some aren’t?

Why does going on a walk help young people feel better?

Why can being on technology have a calming effect?

Why do different students enjoy different subjects at school?

Why is it frustrating for a young person when their perceptions of their lives do not match what others are telling them?

Why are young people's perceptions of whether they have experienced trauma different from the adults around them?

Why might young people with trauma have sensitivities to noise?

Why might young people with trauma have low pain thresholds?

Does a past of trauma have an effect on a young persons taking part in PE?

Why is it that some young people with a history of trauma drink a lot?

Why might outdoor play such as swinging on monkey bars and playing on the swing help a young person with a history of trauma?

Why might colouring help a young person with a history of trauma?

Why do some students find it hard to be in class while some don't?

Why can loud noises make some young people angry, for example lots of people shouting or certain types of music?

Why do some video games make young people with a history of trauma angry or aggressive?

**Home**

What differences can support at home make to the lives of children with difficult backgrounds?

How can schools work in partnership with families?

What are the experiences of adoptive families of children with Education and Health Care Plans?

What are adoptive families' experiences of school behaviour policies?

Why does the relationship between parents and school so often break down, resulting in poor support for the child?

Is homework a good idea for children with a history of trauma or does it cause tension, aggression and violence at home?

What effects does trauma have on the child and the family?

How can we enable traumatised young people to cope with their parents in an educative role (e.g. homework)?

What impact does homework have on stress levels at home?

Is it easy for parents/carers of trauma experienced children to access appropriate, trauma informed support that is also joined up?

Is it common for parents/carers to be blamed for difficulties their TEC have in accessing education?

How prevalent is referral by professionals to parenting courses?

**Interventions & Alternate Provision**

Is PACE (Playfulness, Acceptance, Curiosity, Empathy) therapeutic learning effective to implement in schools?

How do we record outcomes for interventions based on helping children with a history of trauma?

Do forest schools result in better engagement for children with trauma?

What difference can outdoor education make to the lives of children with trauma?

Why is behaviour generally better when students are educated outside?

What is the importance of trauma informed schools & systems in terms of improving outcomes?

What is the importance of trauma informed schools & systems in terms of improving mental health?

What is the importance of trauma informed schools & systems in terms of improving physical health?

What has previous research about Dyadic developmental psychotherapy interventions found about benefit to individual families & whole school Playfulness, Acceptance, Curiosity, Empathy (PACE) informed approaches?

How does the Solihull Approach support trauma informed schools, organisations & homes to improve outcomes?

Why do reward charts not work?

What are behaviour management strategies other than behaviourist/explicit rewards system?

Is Non Violent Resistance (NVR) effective? Why or why not?

What are the benefits and value gained from forest school and how often should forest school happen maximise these benefits?

Is Seguridad an effective intervention?

What is the impact of forest schools, mountain schools, and gardens in education of young people?

How can we give teachers tools to respond to traumatised children when they are dysregulated and display inappropriate behaviour in school?

What works for kids with trauma experience?

What is the impact of early trauma and masking - does this lead to unidentified problems going unaddressed and what strategies can be used to help this?

What are some whole-classroom-trauma responsive approaches?

How effective is the Know Me To Teach Me intervention?

How effective is the Seguridad model of intervention?

What are the barriers to education most commonly seen in trauma experienced children?

What works in terms of overcoming the most common barriers to education?

Why do children under trauma-informed education not get 'told off'. How does this help?

Why is it that a young person having a blanket over their head or being in a dark space can make them feel calmer?

What is the purpose of Zones of Emotional Regulation cards?

Why might being on technology help a young person to be calm?

**Policy and Wider**

Do government academic expectations have an impact on wellbeing?

If so many kids have 'special educational needs' is there a point at which they become 'educational needs' ?

What trauma informed ways of working are there in mainstream education?

What provision exists in mainstream schools for children that cannot regulate their emotions, who run away, freeze on the spot, or fight and retaliate when faced with pressure and difficulties?

What ongoing training do schools provide around trauma informed interventions?

What is the impact of trauma on absenteeism (from school) or illness/symptoms of distress?

What works in helping to mitigate the impact of trauma on absenteeism (from school) or illness/symptoms of distress?

Why doesn't OFSTED allow for PACE (Playfulness, Acceptance, Curiosity, Empathy) training to be carried out in schools?

What does a trauma informed behaviour policy in school look like?

How is trauma informed learning implemented in schools?

Do higher levels of staffing help to support those children who have difficult life stories?

How can evidence based policy & practice-based evidence influence policy to improve grassroots practice?

How does integrated trauma informed school / home working such as Beacon House model improve attendance, wellbeing, social, emotional, and mental health & attainment in kids with complex trauma profiles?

How can we better measure progress (i.e. emotional or academic) in schools?

In what ways do behaviour policies and behaviourist approaches fail children?

How do we provide better alternate provision for those who have experienced trauma and attend a mainstream school?

How can we embed trauma informed approaches in education?

Does a conventional school setting further traumatise children with a history of trauma?

What is the current level of trauma awareness in schools?

What can be done by both primary and secondary schools to reduce the risk of triggering trauma in our children when they move from one into the other?

What is the efficacy of school support measures for Previously Looked After Children- Pupil Premium+, Virtual Schools, Designated Teachers?

Is pupil premium plus used directly for the child it is given for?

How can we make trauma-informed learning effective in mainstream schools?

Can the department of education implement training to ensure all teachers, SEN (special educational needs) staff and teaching assistants are all trauma informed?

Would it be effective to screen children for Adverse Childhood Events (ACE’s) before they start school to ensure they receive the support they need?

How can we measure knowledge and attainment in children who are unable to cope with sitting formal Exams/assessments?

How do schools effectively meet the needs of these children in education, and what needs to change to get there?

How can we provide a curriculum tailored to the needs of children who have complex life stories?

What is the link between sensory considerations provided in schools and funding?

Why is there such variation in practice?

How can we equip schools regarding confidentiality and risk related to information security?

How can we select the right school for the child’s needs?

How can transition between primary to secondary schools be made easier?

How can we support children through transition between schools?

How can families be better supported in cases of home schooling where this is more acceptable than a standard educational setting, and how do we make this a more accessible option?

How can we move away from a curriculum and examination-based approach and provide the child with a more tailored education?

How can we improve training so that teaching can be more trained to deal with children in a more trauma-informed way?

What impact does a trauma informed whole system approach have on outcomes for trauma experienced children?

How widespread is emotionally based school avoidance in trauma experienced children (also called school refusal)?

Has emotionally based school avoidance increased as a result of the pandemic?

What is the role of government and local authorities in holding schools to account with respect to supporting/meeting TEC's needs?

What are local authorities' understanding and application of trauma informed approaches?

Is everyone who has had difficult life experiences, an overthinker?

Do specialist trauma-informed schools reduce the chances of crime/drug use?

Why can’t all children go to a mainstream school? Who decides? Why do they get to decide?

**Schools**

How can we tailor school rules and reward systems in relation to psychologically informed environments and trauma/attachment processes in care experienced children?

How can schools better adapt to meet the needs of children with trauma?

How do schools better meet the needs of children with a trauma history where there is no specific diagnosis?

When a child's behaviour results in exclusion what learning does the school have to avoid this in the future?

If a child needs to escape what escape routes does a school have ?

How can schools make classrooms more trauma informed?

Are children in education receiving a good balance of physical education, drama and creative arts to help them manage their individual trauma and time given to listen to their individual needs?

What is the role of keyworker time & emotionally available adults in the reduction of behaviour that challenges?

Does behaviour led policy in schools compound toxic stress and increase vulnerability, disadvantage, and toxic stress?

Does a trauma informed school have students that are happier, more high achieving, and have good attendance?

How can an attachment focused classroom support child development, behaviour and attainment?

How does current behaviour policy, attendance policy, uniform policy impact education attainment, mental health & attendance for kids with complex life stories?

What is the knowledge of school staff on what trauma is and what its effects on children and adults are?

How is the 'whole child' planned for within schools?

How is equity not equality supported in the classroom setting to maintain a child's right to be supported but not to feel different and for classmates to understand this?

How can we build a trauma informed school community whilst maintaining privacy?

How can we be more mindful of triggers such as Mother’s Day, Father’s Day, birthdays and Christmas, homework, class environment, stigma of adoption, trauma and attachment informed learning?

What is the best way to support children who will respond well to emotional support in school but are determined to keep school and family life separate?

How do we cope when schools claim to be trauma informed but have behavioural policies that contradict with this aim?

How can we make schools emotionally safe?

How can we create an environment that supports trauma informed approaches?

How can we support teaching and support staff to understand complex trauma?

How can support staff use an understanding of complex trauma to form a change in practices?

How can a school support relationships with teachers and other students?

How can schools be positive in their correction of behaviour?

How can we adapt school life and expectations to support children with trauma?

How can professionals work together better to support children with trauma? (How individuals working with the child, teachers, pastoral leaders, social workers, counsellors, support services, Teaching Assistants etc. can communicate and streamline their work.)

What research exists about how we can make the classroom environment better?

How can we prevent children with trauma being labelled as "the naughty child" and give them opportunities for success?

What are experiences of school relationships (parent/school; child/school; child/child)?

What is the level of training within schools for staff to support those who have experienced trauma?

How can relationships at school occur to support children with trauma?

What things about the traditional classroom do not work / are not effective for children affected by trauma?

Do punishments that are not immediate and may appear unlinked to the behaviour contribute to feelings of toxic shame within children with a history of trauma?

Why do children who have experienced trauma find the standard school environments so difficult?

What is the impact of secondary trauma through poor education, lack of support or scaffolding, and school exclusion?

Do teachers in mainstream schools struggle to make whole-school changes to accommodate children who have a history of trauma?

Do examples and follow-ups after trauma-informed training help teachers to implement changes in schools

What is the effect of class make up on support children with a history of trauma receive?

How can schools alter their traditional approaches to better suit teenagers affected by trauma?

Are children with trauma given the same opportunities as other children in mainstream schools? If not why not?

How is sharing specific behaviours that occur in a school crucial to supporting a child holistically?

How can the school address the needs of a child who has experienced trauma within the classroom, without segregating said child from the classroom environment?

How can we monitor trauma-informed accreditation given to schools to ensure that this is not only a 'tick-box' exercise, but supports best practice?

How can we ensure that schools truly understand attachment trauma?

How can we support a school to become an additional secure base for a child?

How can we best support sensory needs to make school a protective factor?

How do we accommodate children who are experiencing attendance problems rather than punishment or placing blame?

Should children who have experienced trauma receive punitive sanctions for their behaviour in school (e.g. isolation, exclusions)?

How is the pupil premium fund used to support the child individually and specifically?

Do reward based systems at school increase feelings of shame and low self-esteem?

Should teaching staff have training on adoption based trauma?

How can we improve information sharing between the child's family and school?

Why don't playgrounds schools provide sensory regulating equipment for all ages?

How can we support children to feel safe in schools?

How can we better aid school staff’s understanding of trauma?

What additional support can be provided by SENCos and related staff for traumatised children?

How can best practice be disseminated within school?

What are the benefits of a key adult in the school acting as an advocate for the child and establishing communication between the school and family?

How can the classroom environment be improved to be less of an overwhelming and unsafe environment?

What adaptations and accommodations do schools make to their behaviour and exclusion policies for TEC?

Why do mainstream schools struggle to accommodate students that aren't "normal"?

How can mainstream schools support all students better?

Why do mainstream schools give detentions for naughty students and how do they feel these benefit their students in the short and long term?

Why do some specialist trauma-informed education schools have a therapy dog, and how might that help the students?

Why don't specialist trauma-informed schools have punishments?

Why do some specialist trauma-informed education schools not have a school uniform or not punish for not wearing a uniform?

**Teachers and Learning**

If education strategies do not work, why is this?

How do teachers live with their own potential trauma?

What alternative communication skills do teachers have that could be trauma informed?

How can schools improve their teaching methods to be more trauma informed?

How can trauma informed learning better engage children with trauma in education?

Do teachers know what trauma informed learning is?

Is an Emotional Literacy Support Assistant (ELSA) effective for supporting the education of children with complex life stories?

Do teachers understand their own reactions to children who act from a place of trauma?

How do early life experiences influence education (e.g. lack of sleep due to parental conflict)?

How do we better understand children and how they learn?

How do we improve trauma-informed learning (as without it kids won't be able to learn)?

How can teachers help children regulate when supporting behaviour?

How does hypervigilance impede learning?

What evidence is there for how we can provide empathetic teaching and support for children and young people with trauma?

How can the curriculum be accessed by children with developmental trauma, and how can it be trauma-informed?

Is Trauma-informed learning part of teacher training and if so in how much detail and if not, why not?

How can we create effective learning environments for children with trauma?

Can children learn when they are triggered by things in the classroom?

To what extent does early life trauma impact language / literacy development?

What are the best ways to boost deficits in the area of language and literacy development?

Is teacher understanding of children with histories of trauma lacking?

Do teachers have high expectations of students that cannot be met?

What is the experience and knowledge of teachers and staff (on trauma, attachment etc)?

How can knowledge of the effects of trauma on learning advise everyday policy and practice?

How does trauma affect children in the classroom? How can staff support these children?

What is the importance or people's experiences of relationships and teaching children with attachment problems?

In what ways is trauma a barrier to learning?

What is the importance of having a tutor as a daily point of contact?

How can we help children feel calm and safe enough in school to learn, including feeling safe around their peers?

How can academic outcomes for children with complex life-stories be improved?

Are teachers & TAs adequately trained & informed to support adopted children?

Why doesn't education in school recognise child developmental stages and use movement and play more in teaching?

What works in terms of supporting these children to access an education?

Dissemination Email

You may remember having taken part in a survey earlier this year about education for children with a difficult life story or trauma. The whole research prioritisation process is now complete and I’m writing to you as you expressed interest in being updated about the results of this exercise.

The process of research prioritisation has two stages. The first, an ‘idea-collecting’ survey, collecting as many questions about education for children with trauma as possible. The second, asking people to vote for the questions they think are most important in this topic area.

We carried out these stages in two different settings: with students at a school which specialises in teaching children with difficult backstories, and nationally with adult stakeholders such as school staff and family members.

In the first round of surveys we heard from six classes at the school who submitted 26 questions in total, and in the adult stakeholder survey 115 submissions which resulted in 221 questions to be included. In the second round of surveys in the school, 8 students completed the task, and there were 47 adult stakeholders who voted on their top priorities. In the adult survey the questions were split up into 6 categories for two purposes: to make them more comparable, and to make them easier to prioritise due to the high number of questions.

The top priorities collected from the students at the school were:

- Is everyone who’s had difficult life experiences, an overthinker?
- Why does being on technology help us be calm

The top priorities of adult stakeholders in each category were:

- Child-Centred: What do trauma responses look like in the classroom?
- Home: How can schools work in partnership in families?
- Interventions and Alternate Provisions: How can we give teachers tools to respond to traumatized children when they are dysregulated and display inappropriate behaviour in school?
- Policy and Wider: What trauma-informed ways of working are there in mainstream education?
- Schools: How do schools better meet the needs of children with a trauma history where there is no specific diagnosis?
- Teaching and Learning: How can schools improve their teaching methods to be more trauma informed?
