# Supplementary material for: What Next for Trauma-Informed Education Research? A Research Prioritisation Exercise with Young People as Informants
Source: J Child Adolesc Trauma. 2025 May 23;18(3):803–13. doi: 10.1007/s40653-025-00711-3 (PMC12433405; doi:10.1007/s40653-025-00711-3)
Supplement: Supplementary file 7 — Supplementary file7 (DOCX 26 KB) [file 40653_2025_711_MOESM7_ESM.docx]

**What next for trauma-informed education research? A research prioritisation exercise with young people as informants.**

**Research Prioritisation Summary –**
**Education for Children with Trauma / Complex Life Stories**

This document breaks down the top question for each group that participated in ranking the second questionnaire, and where these top ranked questions came overall for that category.

**If you would like further information please contact: [xxx]**

| **Group** | **Top Question** | **Place Ranked overall** |
| --- | --- | --- |
| **Child-Centred** | | |
| Professional | 2.4. How do we give children who have complex needs a voice? | 4 |
| Family | 2.12. What do trauma responses look like in the classroom? | 1 |
| Other | 2.2. How does the learning from trauma informed training in a school translate into the lived experience for a child/young person?  2.4. How do we give children who have complex needs a voice?  2.12. What do trauma responses look like in the classroom? | 3  4  1 |
| **Home** |  |  |
| Professional | 3.2. How can schools work in partnership with families? | 1 |
| Family | 3.4. What are adoptive families' experiences of school behaviour policies? | 2 |
| Other | 3.2. How can schools work in partnership with families?  3.5. Why does the relationship between parents and school so often break down, resulting in poor support for the child? | 1  Not top 5 |
| **Interventions** |  |  |
| Professional | 4.7. What is the importance of trauma informed schools & systems in terms of improving mental health? | 3 |
| Family | 4.17. How can we give teachers tools to respond to traumatised children when they are dysregulated and display inappropriate behaviour in school? | 1 |
| Other | 4.17. How can we give teachers tools to respond to traumatised children when they are dysregulated and display inappropriate behaviour in school? | 1 |
| **Policy & Wider** |  |  |
| Professional | 5.10. How is trauma informed learning implemented in schools? | 4 |
| Family | 5.3. What trauma informed ways of working are there in mainstream education? | 1 |
| Other | 5.9. What does a trauma informed behaviour policy in school look like? | 2 |
| **School** |  |  |
| Professional | 6.1. How can we tailor school rules and reward systems in relation to psychologically informed environments and trauma/attachment processes in care experienced children?  6.8. What is the role of keyworker time & emotionally available adults in the reduction of behaviour that challenges? | 3 |
| Family | 6.3. How do schools better meet the needs of children with a trauma history where there is no specific diagnosis? | 1 |
| Other | 6.2. How can schools better adapt to meet the needs of children with trauma? | 2 |
| **Teachers & Learning** |  |  |
| Professional | 7.8. Do teachers understand their own reactions to children who act from a place of trauma? | 2 |
| Family | 7.4. How can schools improve their teaching methods to be more trauma informed? | 1 |
| Other | 7.4. How can schools improve their teaching methods to be more trauma informed? | 1 |
